# Supplementary material for: Photobiocatalytic synthesis of chiral secondary fatty alcohols from renewable unsaturated fatty acids
Source: Nat Commun. 2020 May 7;11:2258. doi: 10.1038/s41467-020-16099-7 (PMC7206127; doi:10.1038/s41467-020-16099-7)
Supplement: Supplementary file 1 — Supplementary Information [file 41467_2020_16099_MOESM1_ESM.pdf]

Supplementary Information

Photobiocatalytic synthesis of chiral secondary fatty alcohols from renewable unsaturated fatty acids

Zhang et al

Supplementary Figures

A

MYYSNGNYEAFARPRKPAGVDKKHAYIVGAGLAGLSAAVFLIRDAQMPGENIHIFEELPIAGGSLDGQDRPDVGFVTRGGREM
ENHFECMWDMYRSIPSLEIPGASYLDEYYWLDKDDPNSSNCRLTYNRGNEVPTDGKYLLGKSTKELMKLILTPEDQLGDLTIGDY
FSEDFFKSNFWIYWSTMFAFEKWHSLAEMRRYAMRFIHHIDGLPDFTALKFNKYNQYESMTKPLLAYLKDHGVKFEYDTQVQN
VLVDTKNGEKHAKKIILKQGGEDKTINLTDDDLVFVTNGSITESSNYGSHHQVAQPTRALGGSWKLWENIAKQSPAFGHPDVFC
KNIPNRSWFISATATVKNSQIEPYIERLTKRDLHDGKVNTGGIITVTDSNWMMSWTIHRQPHFKSQKPNETIVWIYGLYSDTEG
NYIKKRIVDCTGEEITKEWLYHLGVPEALIDDLAKEESVNTVPVYMPFVTSYFMPRVKGDRPDVVPEGSANLAFIGNFAESPTRD
TVFTTEYSVRTAMEAVYSLLNVDRGVPEVFNSIYDIRELMRAMYYMNDKKPLEEMDLPIPKIVEKPLLKKIKKNWIGELMEEQHL
L

B

ATGTACTATTCGAACGGGAACTATGAAGCTTTTGCTCGTCCTCGTAAGCCAGCGGGGGTTGACAAAAAGCATGCATATATT
GTCGGTGCTGGTTTAGCAGGCCTTTCTGCGGCTGTTTTCTTAATTCGGGATGCTCAAATGCCTGGAGAGAATATCCATATTT
TTGAAGAATTACCAATTGCTGGTGGTTCACTCGATGGTCAAGATCGCCCTGATGTTGGATTTGTTACTCGTGGCGGCCGTG
AAATGGAAAACCACTTTGAATGTATGTGGGACATGTATCGTTCAATTCCATCTTTGGAAATCCCGGGAGCATCTTATCTCGA
TGAATATTATTGGTTAGACAAGGATGATCCAAACAGTTCAAATTGTCGCTTAACTTATAACCGGGGCAATGAGGTTCCTAC
TGACGGCAAGTATCTTCTTGGAAAGTCAACTAAGGAATTGATGAAACTGATCCTTACACCTGAAGACCAGCTCGGCGACTT
AACGATCGGTGATTACTTCTCTGAGGACTTCTTCAAGAGTAATTTCTGGATTTACTGGTCAACCATGTTTGCTTTTGAAAAA
TGGCATTCGCTAGCAGAAATGCGGCGATACGCAATGCGATTTATCCACCACATTGACGGTTTGCCTGACTTTACTGCTCTTA
AATTCAATAAGTACAACCAGTATGAATCAATGACCAAACCATTGCTTGCATACCTTAAAGATCATGGTGTTAAGTTTGAATA
CGATACCCAGGTTCAAAACGTCCTAGTTGATACCAAGAATGGTGAAAAGCACGCTAAAAAGATTATTCTTAAGCAAGGCG
GCGAAGATAAAACGATCAACTTAACTGACGATGATCTGGTATTTGTTACTAATGGTTCAATCACGGAAAGTTCAAATTATG
GGAGTCATCACCAGGTAGCACAACCAACTCGTGCCCTTGGCGGTAGCTGGAAATTATGGGAGAACATTGCTAAACAATCG
CCAGCATTTGGTCATCCAGATGTCTTTTGTAAAAACATTCCTAACCGCAGTTGGTTCATTTCTGCAACTGCGACCGTTAAGA
ATTCACAAATTGAACCGTACATTGAACGACTAACTAAGCGTGATCTTCATGATGGAAAGGTAAACACTGGTGGAATTATCA
CCGTCACTGATTCTAATTGGATGATGAGTTGGACCATCCACCGGCAACCTCATTTTAAGAGTCAAAAGCCAAATGAAACCA
TTGTGTGGATCTATGGTCTCTATTCTGATACAGAAGGTAATTACATTAAGAAACGAATTGTTGATTGTACTGGGGAAGAAA
TTACTAAAGAATGGCTCTACCATCTCGGTGTTCCGGAAGCTTTAATTGACGATTTAGCAAAAGAAGAGTCGGTTAATACCG
TCCCTGTTTATATGCCATTCGTTACTAGCTACTTTATGCCGCGGGTAAAAGGCGATCGTCCTGATGTTGTTCCTGAGGGGTC
AGCTAACTTGGCCTTCATTGGTAACTTTGCCGAATCACCAACTCGGGATACCGTCTTTACAACTGAATATTCAGTCCGGACG
GCAATGGAAGCTGTTTATAGTCTCCTCAACGTTGATCGCGGGGTACCAGAAGTCTTTAATTCGATCTATGATATTCGCGAAT
TAATGCGGGCAATGTACTATATGAATGATAAAAAGCCACTTGAAGAAATGGATCTGCCAATTCCAAAGATTGTTGAGAAA
CCACTGCTCAAGAAAATTAAGAAGAATTGGATTGGCGAATTAATGGAAGAACAGCATTTACTTTAA

Supplementary Figure 1. Aminoacid aquence (A) and DNA sequence (B) of the recombinant *Lr*OH from *Lactobacillus reuteri*.

ATGAAATCTTCTCACCATCACCATCACCATGGTTCTTCTATGAGCGATAAAATTATTCACCTGACTGACGACAGTTTTGACAC
GGATGTACTCAAAGCGGACGGGGCGATCCTCGTCGATTTCTGGGCAGAGTGGTGCGGTCCGTGCAAAATGATCGCCCCG
ATTCTGGATGAAATCGCTGACGAATATCAGGGCAAACTGACCGTTGCAAAACTGAACATCGATCAAAACCCTGGCACTGC
GCCGAAATATGGCATCCGTGGTATCCCGACTCTGCTGCTGTTCAAAAACGGTGAAGTGGCGGCAACCAAAGTGGGTGCAC
TGTCTAAAGGTCAGTTGAAAGAGTTCCTCGACGCTAACCTGGCCGGGATCGAGGAAAACCTGTACTTCCAATCCGCGTCTG
CCGTTGAAGACATCCGTAAAGTCCTGTCCGATTCTTCGTCTCCGGTGGCGGGTCAGAAATATGACTACATCCTGGTTGGCG
GTGGCACCGCGGCGTGCGTGCTGGCAAACCGTCTGAGCGCTGACGGTTCCAAACGTGTACTGGTTCTGGAAGCAGGCCC
GGATAACACCTCCCGCGACGTTAAGATTCCGGCGGCGATCACCCGCCTGTTCCGCTCCCCGCTGGACTGGAACCTGTTCTC
TGAACTGCAGGAACAGCTTGCGGAACGTCAGATCTACATGGCGCGTGGCCGTCTGCTGGGCGGTTCCAGCGCGACTAACG
CCACTCTGTACCACCGTGGTGCGGCGGGTGATTACGACGCATGGGGTGTTGAAGGCTGGTCCAGCGAAGACGTTCTGTCT
TGGTTCGTCCAGGCGGAAACCAACGCGGACTTCGGTCCGGGCGCTTATCATGGCAGCGGCGGCCCGATGCGTGTGGAAA
ACCCGCGTTACACCAACAAACAGCTGCACACTGCTTTCTTCAAGGCTGCTGAAGAAGTTGGTCTTACCCCGAACTCCGATTT
CAACGATTGGAGCCATGACCACGCCGGTTACGGCACCTTTCAGGTGATGCAGGATAAAGGCACCCGCGCGGATATGTACC
GTCAGTATCTGAAACCTGTGCTGGGTCGTCGCAACCTGCAGGTACTGACCGGCGCTGCAGTGACCAAAGTCAACATCGAC
CAGGCTGCGGGCAAAGCGCAGGCTCTGGGTGTTGAATTCTCCACCGACGGCCCAACCGGCGAACGCCTGTCTGCGGAACT
GGCTCCGGGTGGTGAGGTCATCATGTGCGCAGGTGCTGTTCACACCCCGTTCCTGCTGAAACATTCCGGCGTTGGCCCGTC
TGCTGAGCTGAAAGAATTCGGCATCCCGGTTGTTAGCAACCTGGCTGGTGTTGGCCAGAACCTGCAGGATCAGCCGGCGT
GCCTGACCGCGGCTCCGGTTAAAGAAAAATACGACGGTATTGCCATTTCTGATCACATCTACAACGAAAAAGGCCAGATCC
GTAAACGTGCAATCGCATCCTACCTGCTGGGTGGTCGTGGCGGTCTGACTTCCACCGGTTGCGATCGCGGTGCCTTCGTTC
GTACCGCGGGTCAGGCGCTGCCGGACCTGCAGGTTCGCTTCGTTCCAGGTATGGCGCTGGACCCGGACGGTGTTAGCACC
TACGTTCGTTTTGCTAAATTCCAGAGCCAGGGTCTGAAATGGCCGAGCGGCATCACCATGCAGCTGATCGCTTGCCGTCCG
CAGTCTACCGGCTCCGTCGGTCTTAAATCCGCTGACCCGTTTGCGCCGCCGAAACTGTCACCAGGTTACCTGACCGACAAA
GACGGTGCTGATCTGGCTACCCTGCGTAAAGGCATCCATTGGGCACGTGATGTTGCGCGTAGCTCTGCTCTGTCCGAATAC
CTGGATGGTGAGCTGTTCCCAGGTAGCGGCGTTGTTTCTGATGATCAGATCGATGAATATATCCGTCGTTCTATCCACTCGT
CCAACGCTATCACTGGCACCTGTAAAATGGGTAACGCAGGTGACAGCAGCTCTGTGGTAGACAACCAGCTGCGTGTTCAC
GGTGTTGAAGGCCTGCGCGTTGTTGACGCTAGCGTTGTTCCGAAAATTCCGGGTGGTCAGACCGGTGCGCCGGTAGTTAT
GATCGCTGAACGCGCAGCAGCTCTGCTGACGGGGAAAGCAACCATTGGTGCATCTGCTGCTGCACCGGCGACCGTAGCTG
CATAA

Supplementary Figure 2. Sequence of *Cv*FAP from *Chlorella variabilis* NC64A.

A

MGEDKETNILAGLGNTISQVENVVAASLRPLPTATGDGTYVAESTQTGLAKDLSHVDLKDVRTLAEVVKSAATGEPVDDKQYIM
ERVIQLAAGLPSTSRNAAELTKSFLNMLWNDLEHPPVSYLGADSMHRKADGSGNNRFWPQLGAAGSAYARSVRPKTMQSPSL
PDPETIFDCLLRRKEYREHPNKISSVLFYLASIIIHDLFQTDPKDNSVSKTSSYLDLSPLYGNNQDEQNLVRTFKDGKLKPDCFATKR
VLGFPPGVGVLLIMFNRFHNYVVDQLAAINECGRFTKPDESNVDEYAKYDNNLFQTGRLVTCGLYANIILKDYVRTILNINRTDST
WSLDPRMEMKDGLLGEAAAMATGNQVSAEFNVVYRWHACISKRDEKWTEDFHREIMPGVDPSTLSMQDFVAGLGRWQA
GLPQEPLERPFSGLQRKPDGAFNDDDLVNLFEKSVEDCAGAFGASHVPAIFKSVEALGIMQARRWNLGTLNEFRQYFNLAPHK
TFEDINSDPYIADQLKRLYDHPDLVEIYPGVVVEEAKDSMVPGSGLCTNFTISRAILSDAVALVRGDRFYTVDYTPKHLTNWAYNE
IQPNNAVDQGQVFYKLVLRAFPNHFDGNSIYAHFPLVVPSENEKILKSLGVAEKYSWEKPSRISHPIFISSHAACMSILENQETFKV
TWGRKIEFLMQRDKHQYGKDFMLSGDRPPNAASRKMMGSALYRDEWEAEVKTFYEQTTLKLLHKNSYKLAGVNQVDIVRDV
ANLAQVHFCSSVFSLPLKTDSNPRGIFAESELYKIMAAVFTAIFYDADIGKSFELNQAARTVTQQLGQLTMANVEIIAKTGLIANLV
NRLHRRDVLSEYGIHMIQRLLDSGLPATEIVWTHILPTAGGMVANQAQLFSQCLDYYLSEEGSGHLPEINRLAKENTPEADELLT
RYFMEGARLRSSVALPRVAAQPTVVEDNGEKLTIKAGQVVMCNLVSACMDPTAFPDPEKVKLDRDMNLYAHFGFGPHKCLGL
DLCKTGLSTMLKVLGRLDNLRRAPGAQGQLKKLSGPGGIAKYMNEDQSGFTPFPSTMKIQWDGELPQLKEDF

B

ATGGGTGAAGACAAAGAAACAAATATCCTCGCCGGCCTCGGAAACACCATTTCCCAAGTAGAAAACGTTGTTGCGGCATC
GTTACGACCTTTGCCAACGGCAACGGGTGATGGAACCTACGTTGCCGAATCCACTCAGACGGGCTTGGCCAAAGATCTGA
GCCATGTCGACCTCAAGGATGTCCGCACACTCGCCGAAGTCGTCAAGAGTGCGGCTACGGGAGAGCCGGTTGATGACAA
GCAGTATATCATGGAAAGAGTGATTCAGTTAGCTGCTGGCTTACCATCGACATCTCGCAACGCTGCAGAGCTAACCAAGTC
ATTTTTGAACATGCTGTGGAATGACTTGGAACATCCACCAGTTTCTTATCTAGGAGCTGATTCTATGCACCGCAAAGCCGAC
GGCTCGGGTAATAATCGTTTCTGGCCTCAACTTGGCGCTGCTGGTAGCGCGTACGCAAGATCTGTTCGGCCCAAGACGAT
GCAGTCTCCATCCCTGCCCGATCCTGAGACTATTTTCGATTGCCTGCTCCGCCGGAAAGAGTACAGGGAGCATCCTAATAA
GATATCAAGCGTTCTATTCTACCTCGCTTCAATCATTATTCATGACCTATTCCAGACAGACCCTAAAGATAATTCCGTGTCCA
AGACATCGTCATATTTGGACCTCTCACCTTTGTATGGCAATAATCAAGACGAGCAGAACCTTGTTCGTACGTTCAAGGATG
GAAAGCTTAAGCCAGATTGTTTCGCTACCAAGCGAGTGTTGGGCTTTCCTCCCGGCGTCGGCGTTCTACTGATCATGTTCA
ACCGCTTCCACAACTATGTGGTTGATCAATTGGCGGCGATCAACGAATGCGGCCGATTCACCAAACCTGACGAGTCCAACG
TTGACGAGTATGCTAAATACGATAACAATCTCTTCCAAACCGGGCGACTGGTGACTTGTGGGTTGTACGCAAATATTATCC
TAAAAGATTATGTCCGAACGATTTTGAATATAAACCGGACAGATAGCACCTGGAGTTTGGACCCCAGAATGGAAATGAAG
GATGGTTTATTAGGTGAAGCAGCAGCAATGGCAACCGGGAACCAGGTGTCAGCCGAATTTAATGTCGTGTACCGGTGGCA
CGCTTGCATTTCTAAGCGCGATGAAAAATGGACAGAGGATTTTCACCGTGAAATCATGCCGGGAGTGGATCCAAGCACAC
TATCGATGCAAGATTTTGTCGCGGGTCTTGGACGGTGGCAGGCAGGACTCCCACAAGAGCCACTTGAGCGCCCATTCTCT
GGCTTACAGCGTAAGCCGGACGGTGCATTCAACGACGATGACCTGGTTAATCTGTTTGAGAAGAGTGTTGAAGACTGCGC
AGGTGCATTTGGTGCGTCTCACGTTCCAGCCATCTTCAAGAGCGTTGAAGCTCTCGGGATAATGCAGGCTCGGAGATGGA
ACTTGGGAACGCTCAATGAGTTCCGCCAATATTTCAATCTGGCTCCTCATAAGACCTTTGAGGATATCAACTCCGATCCGTA
CATTGCGGATCAGCTCAAGCGGCTGTATGATCATCCAGATCTTGTGGAGATTTACCCTGGTGTTGTTGTGGAAGAAGCCAA
AGACTCCATGGTCCCTGGAAGCGGCCTTTGCACGAACTTCACTATATCCAGGGCAATCCTTTCGGATGCGGTGGCATTGGT
TCGCGGTGATAGATTTTACACTGTCGACTACACTCCGAAGCACCTTACGAATTGGGCCTACAACGAGATTCAGCCTAACAA
CGCCGTCGATCAAGGTCAGGTATTCTACAAGCTGGTTCTTCGCGCATTCCCAAACCATTTTGATGGAAATTCTATCTATGCT
CATTTCCCCCTTGTCGTTCCCTCGGAAAATGAGAAAATATTGAAGAGCCTTGGGGTTGCCGAGAAGTATAGCTGGGAAAA
GCCCAGTCGTATCTCTCATCCGATTTTCATCAGCTCTCATGCCGCGTGCATGTCCATCCTCGAAAATCAAGAAACGTTCAAG
GTGACTTGGGGTAGGAAGATTGAGTTCCTTATGCAACGCGATAAGCACCAATACGGGAAGGACTTCATGCTGTCTGGAGA
CCGGCCACCCAACGCTGCATCGCGCAAGATGATGGGTTCCGCCTTGTATCGCGATGAATGGGAGGCTGAGGTCAAAACTT
TCTACGAGCAAACAACTCTAAAACTCTTGCATAAGAACTCCTACAAACTTGCGGGCGTTAACCAAGTCGATATCGTTCGTG
ATGTGGCCAATCTCGCCCAAGTCCACTTCTGCTCTAGCGTCTTCTCATTGCCACTGAAAACAGACTCTAATCCTAGGGGTAT

CTTCGCAGAGTCGGAACTGTACAAGATAATGGCTGCAGTTTTCACTGCCATCTTCTACGACGCAGATATTGGGAAATCGTT
CGAGCTAAACCAGGCCGCCCGTACTGTAACGCAGCAGCTGGGCCAGCTAACTATGGCCAACGTCGAGATCATAGCCAAAA
CCGGCTTGATCGCTAACCTCGTGAACCGCCTTCACCGGCGCGACGTGCTTAGCGAATATGGCATCCATATGATCCAGCGTC
TACTGGATAGTGGTCTCCCAGCGACAGAGATTGTATGGACTCATATCCTTCCTACGGCCGGTGGAATGGTGGCAAACCAA
GCACAACTGTTTTCGCAATGTCTGGACTATTATCTCTCGGAAGAGGGCTCTGGGCATCTTCCTGAGATCAACCGACTGGCC
AAGGAAAATACCCCGGAAGCTGATGAGCTACTTACACGCTATTTCATGGAAGGTGCTCGGCTACGGTCATCCGTTGCCCTG
CCTCGAGTAGCTGCGCAGCCCACGGTCGTAGAAGACAATGGCGAAAAACTCACCATCAAAGCTGGCCAGGTCGTTATGTG
TAACCTGGTCTCTGCGTGCATGGATCCTACTGCCTTTCCAGATCCAGAGAAGGTCAAACTTGACCGTGACATGAACTTATAC
GCCCACTTTGGCTTTGGGCCCCACAAGTGTTTGGGCTTAGACCTATGCAAGACAGGGCTGAGCACGATGCTAAAAGTACTT
GGACGCTTGGACAATCTCCGTCGTGCTCCTGGAGCGCAGGGACAGTTGAAGAAGCTTTCTGGACCTGGCGGGATCGCTAA
GTATATGAATGAGGATCAAAGCGGCTTCACTCCCTTCCCGTCAACTATGAAGATCCAATGGGACGGCGAATTGCCCCAACT
GAAGGAAGATTTT

Supplementary Figure 3. Amino acid sequence (A) and DNA sequence (B) of the 5,8-diol synthase from *Aspergillus nidulans*, *An*DS.

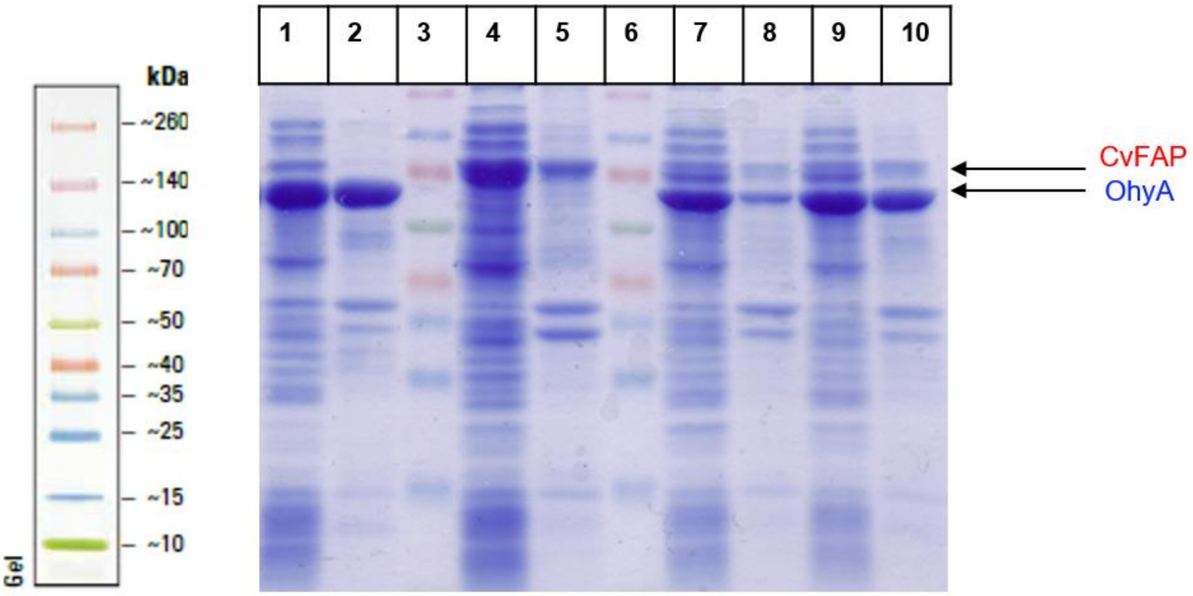

Supplementary Figure 4. SDS-PAGE analysis of the proteins expressed in the recombinant *E. coli* cells. Lanes 1 and 2 show the proteins expressed in the recombinant *E. coli* BL21(DE3) pACYC-PelBSS-OhyA. Lanes 4 and 5 show the proteins expressed in the recombinant *E. coli* BL21(DE3) pET28a-*Cv*FAP. Lanes Lanes 7 to 10 show the proteins expressed in the recombinant *E. coli* BL21(DE3) pACYC-PelBSS-OhyA/pET28a-*Cv*FAP. The gene expression was induced by 0.1 mM IPTG in the cases shown in the lanes 1, 2, 7, and 8, whereas induced by 0.5 mM IPTG in the cases shown in the lanes 4, 5, 9, and 10. The lanes 1, 4, 7, and 9 show soluble fraction of the protein extracts, while the lanes 2, 5, 8, 10 show the insoluble fraction. The biotransformation shown in Figure 6 was carried out by the recombinant *E. coli* BL21(DE3) pACYC-PelBSS-OhyA/pET28a-*Cv*FAP after induction of the gene expression with 0.5 mM IPTG.

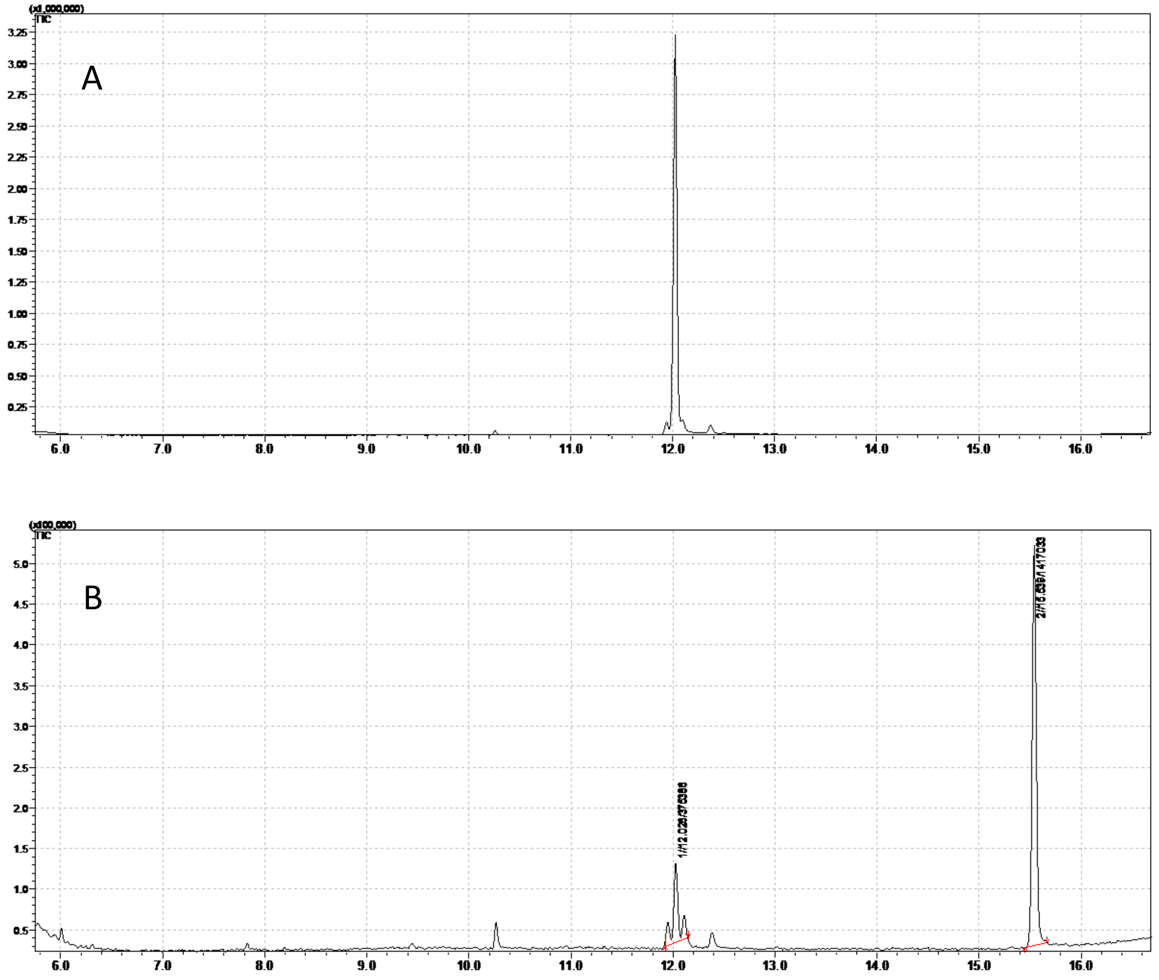

Supplementary Figure 5. GC chromatograms of control reactions using *E. coli* with empty vectors. The hydroxylation, decarboxylation and dihydroxylation reactions of oleic acid using *Lr*OH (A, hydroxylated product should appear at 15.52 min), *Cv*FAP (B), decarboxylated product should appear at 9.25 min).

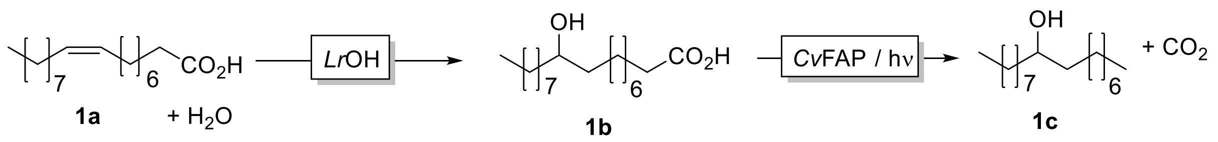

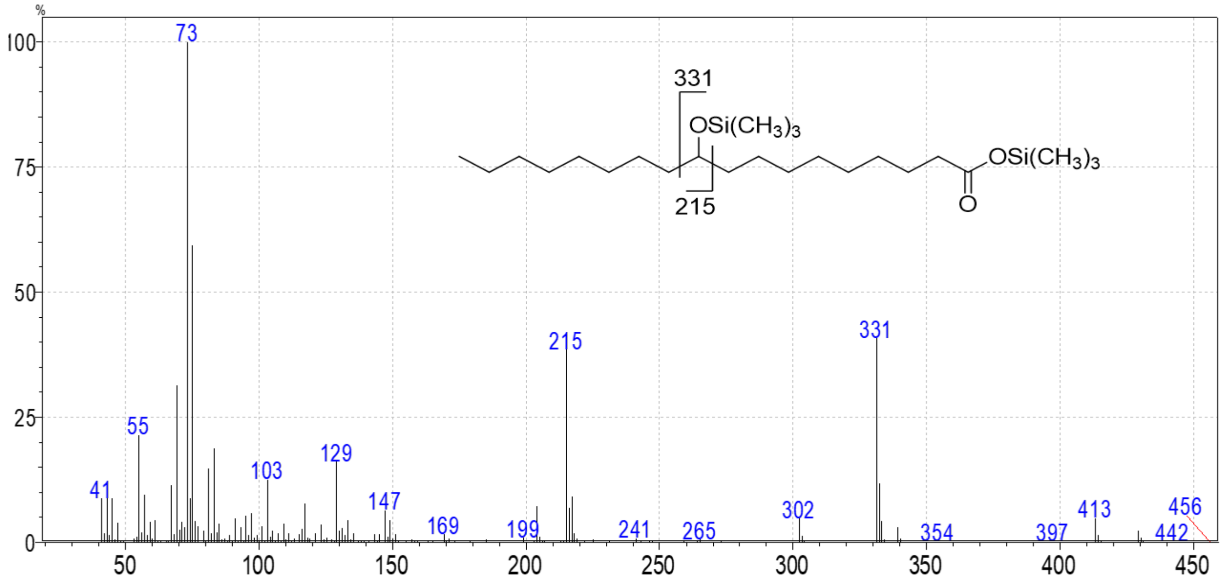

Supplementary Figure 6. GC-MS spectrum of product **1b**.

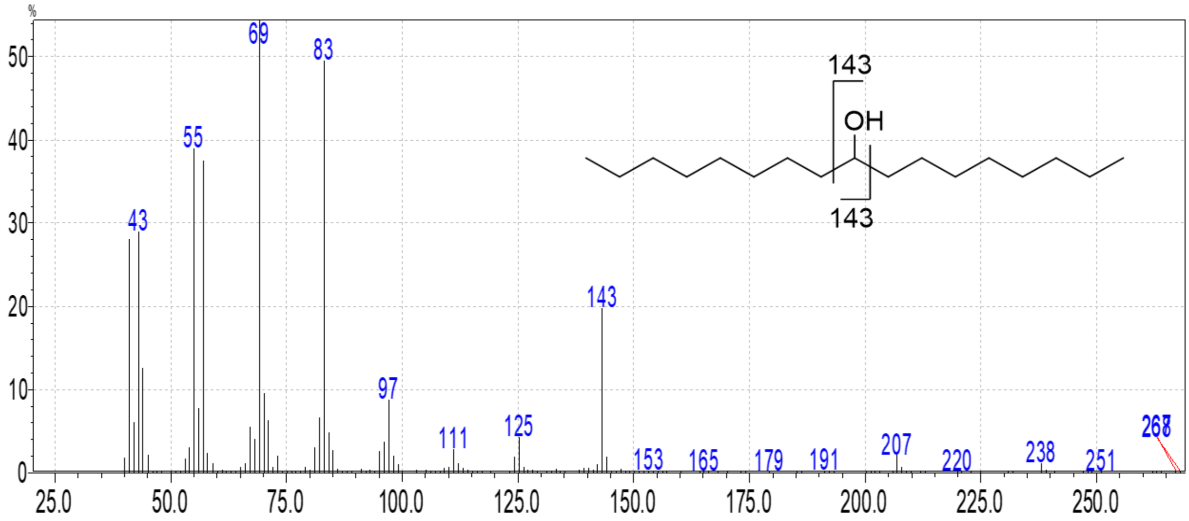

Supplementary Figure 7. GC-MS spectrum of product **1c**.

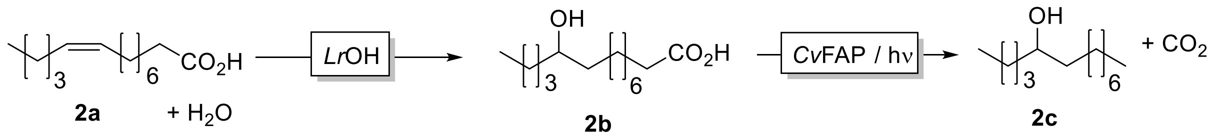

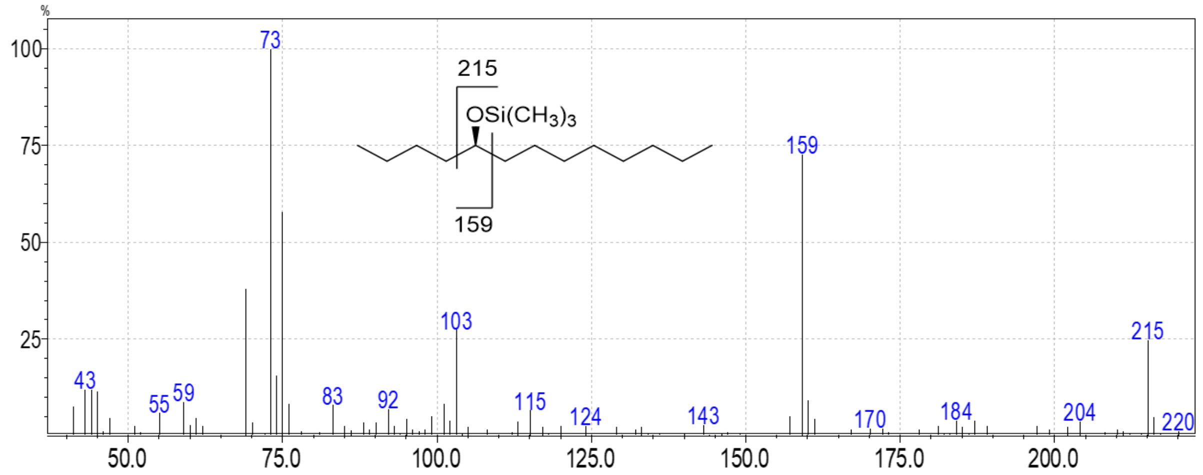

Supplementary Figure 8. GC-MS spectrum of product **2c**.

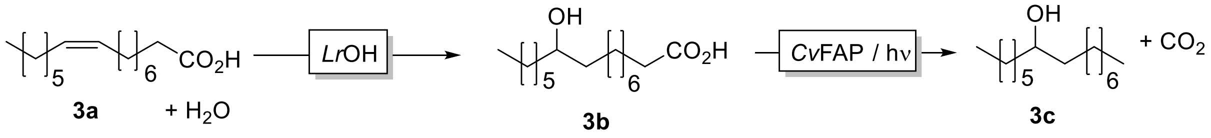

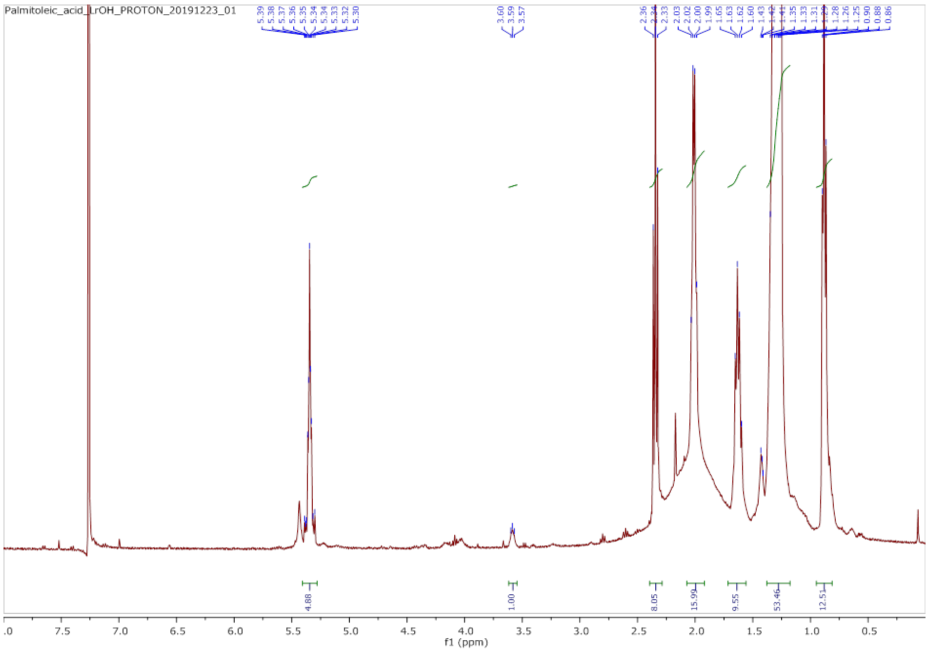

Supplementary Figure 9. ^{1}H NMR spectrum of 10-hydroxyhexadecanoic acid (**3b**).

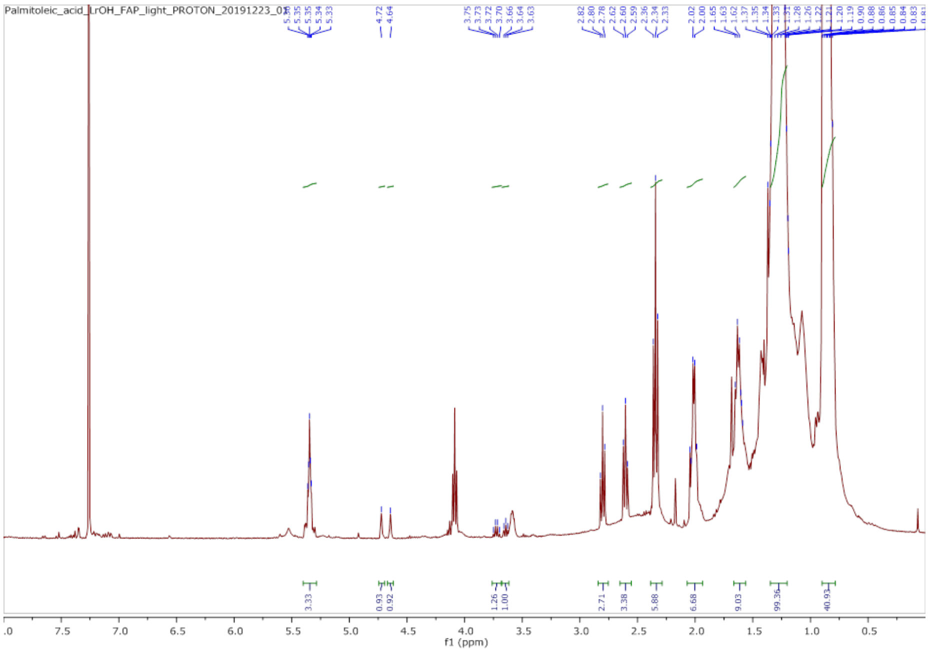

Supplementary Figure 10. ^{1}H NMR spectrum of (*R*)-pentadecan-7-ol (**3c**).

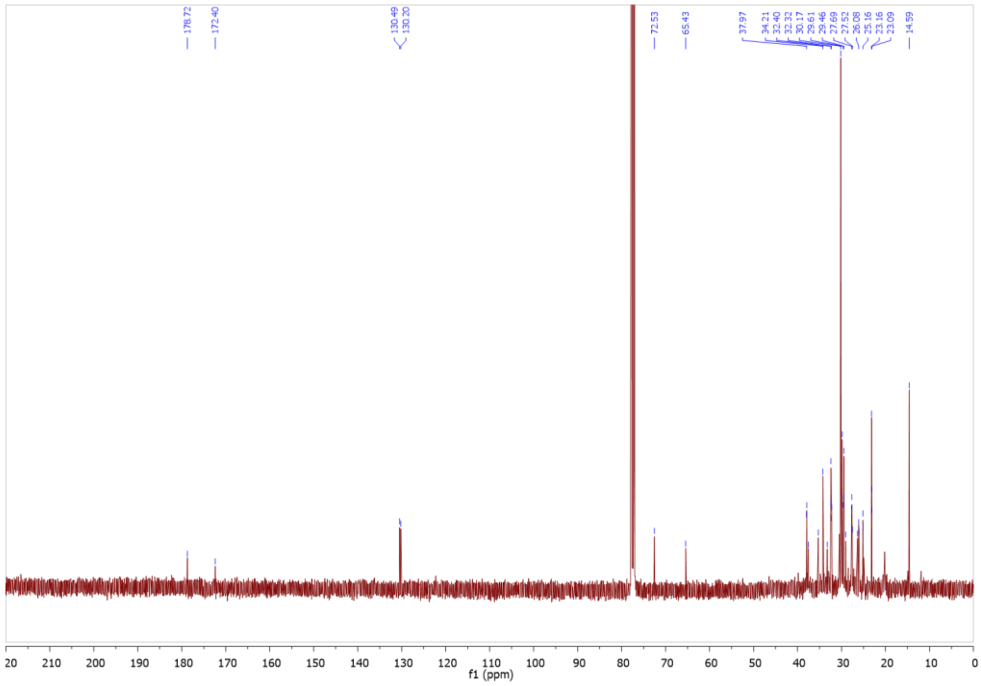

Supplementary Figure 11. ^{13}C NMR spectrum of (*R*)-pentadecan-7-ol **(3c).**

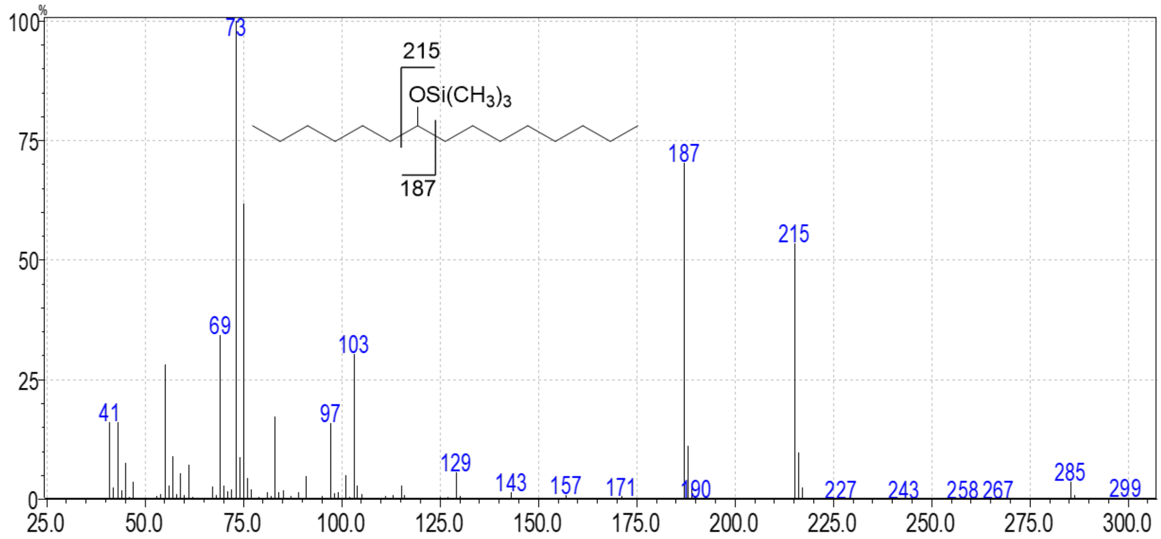

Supplementary Figure 12. GC-MS analysis showing mass spectrum of silylated product (**3c**).

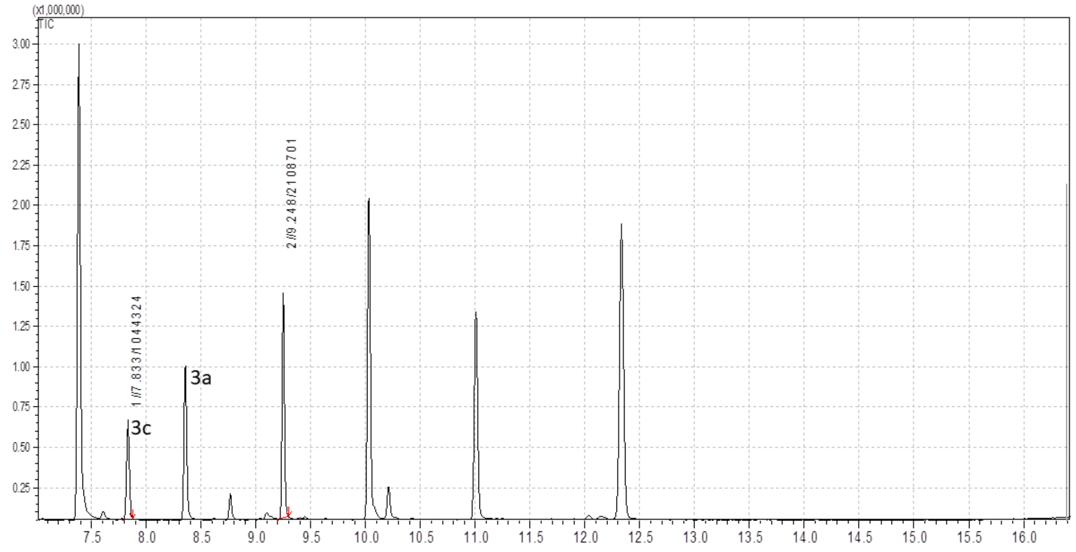

Supplementary Figure 13. Exemplary GC chromatogram of the reaction mixture after silylation (BSTFA) Retention times: **3c**: 7.83 min, **3a**: 8.76 min, 9-heptadecanol: 9.25 min.

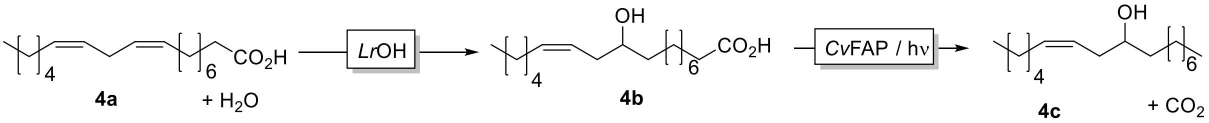

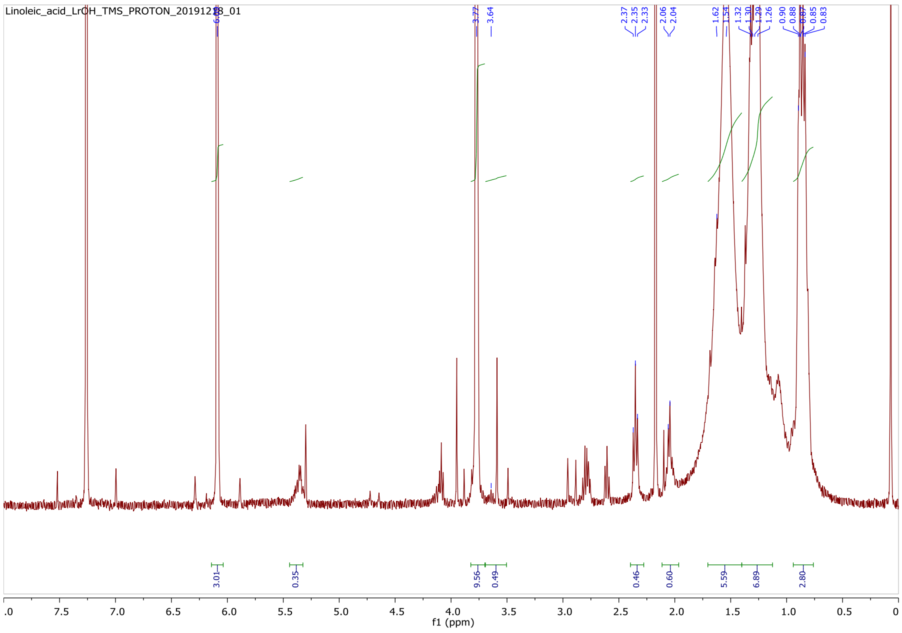

Supplementary Figure 14. ^{1}H NMR spectrum of (*Z*)-10-hydroxyoctadec-12-enoic acid (**4b**).

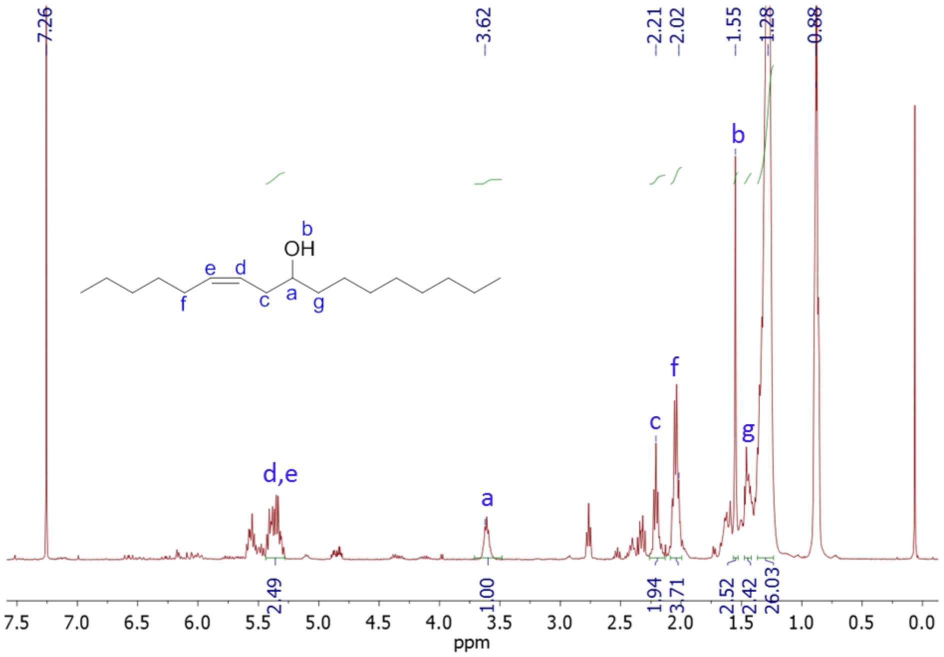

Supplementary Figure 15. ^{1}H NMR spectrum of (*Z*)-heptadec-6-en-9-ol (**4c**).

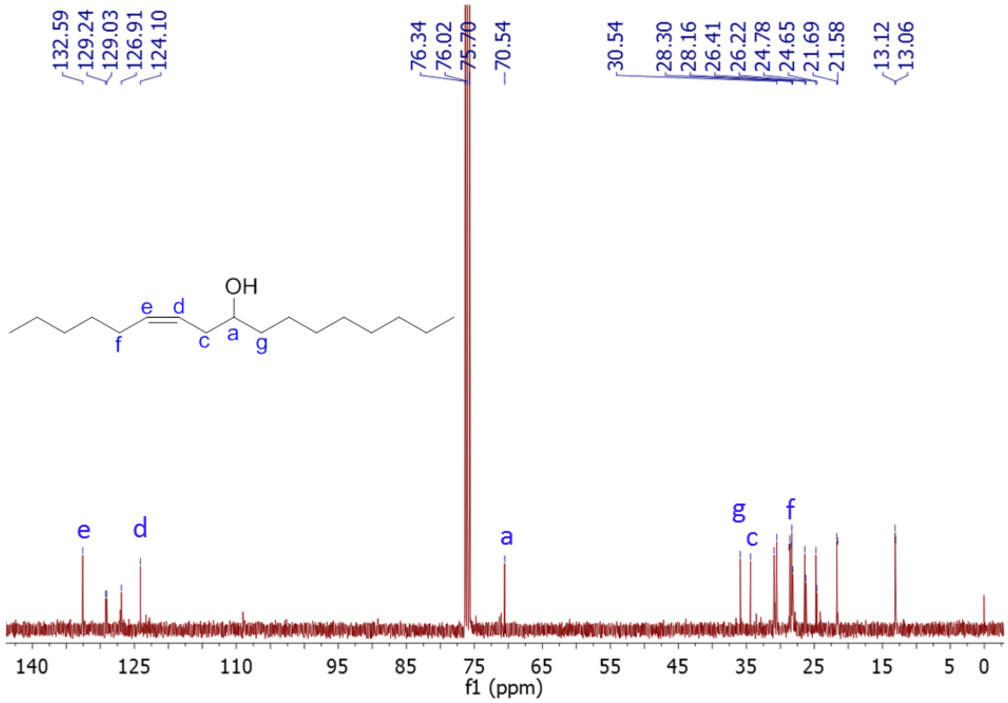

Supplementary Figure 16. ^{13}C NMR spectrum of (*Z*)-heptadec-6-en-9-ol (**4c**).

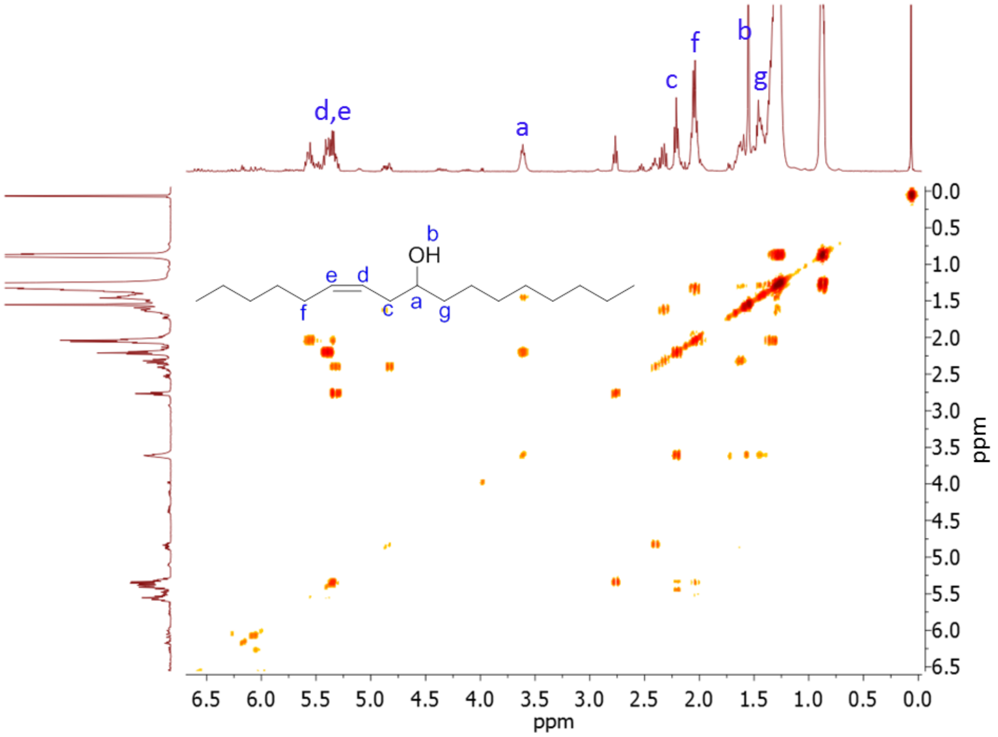

Supplementary Figure 17. gCOSY spectrum of (*Z*)-heptadec-6-en-9-ol (**4c**).

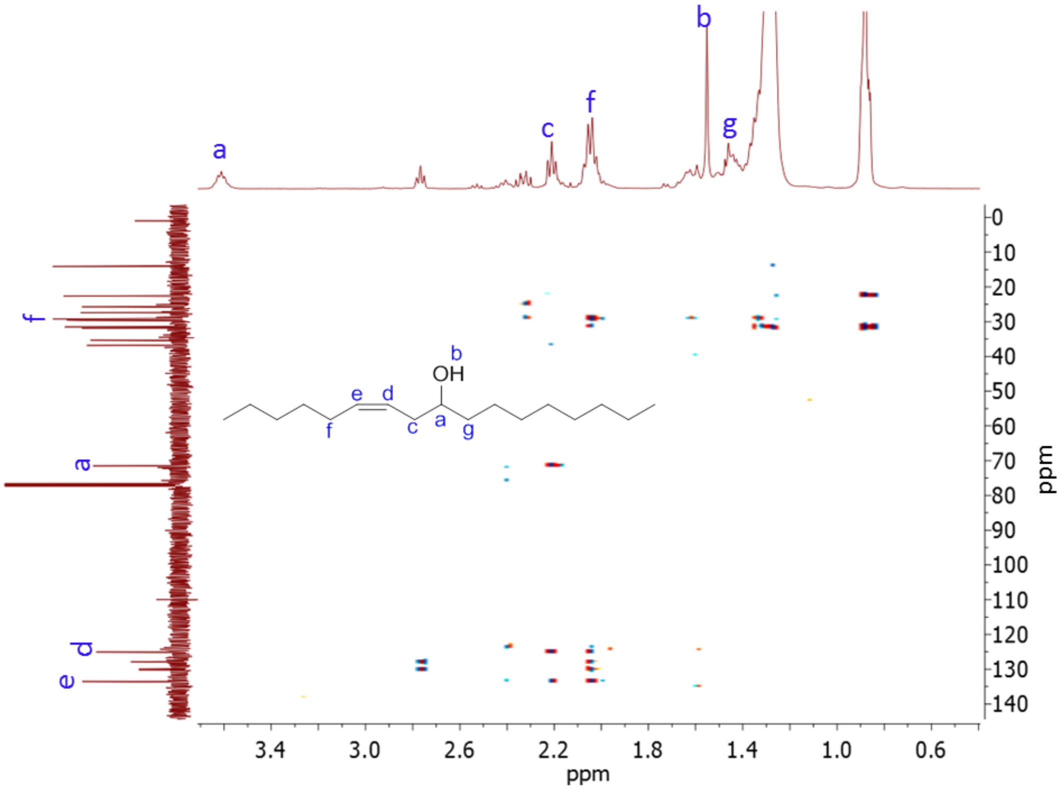

Supplementary Figure 18. gHMQC spectrum of (*Z*)-heptadec-6-en-9-ol (**4c**).

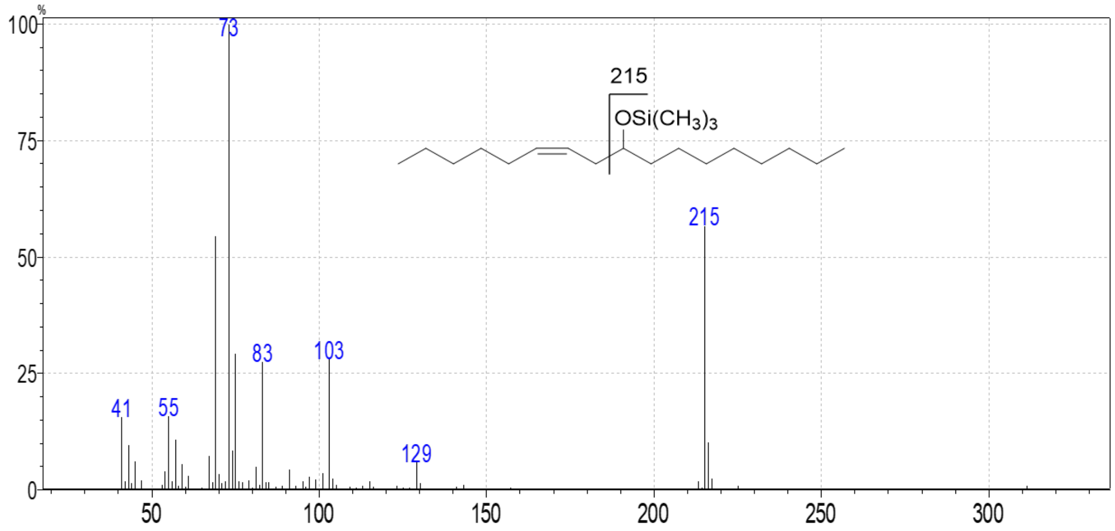

Supplementary Figure 19. GC-MS analysis showing mass spectrum of silylated (*Z*)-heptadec-6-en-9-ol (**4c**).

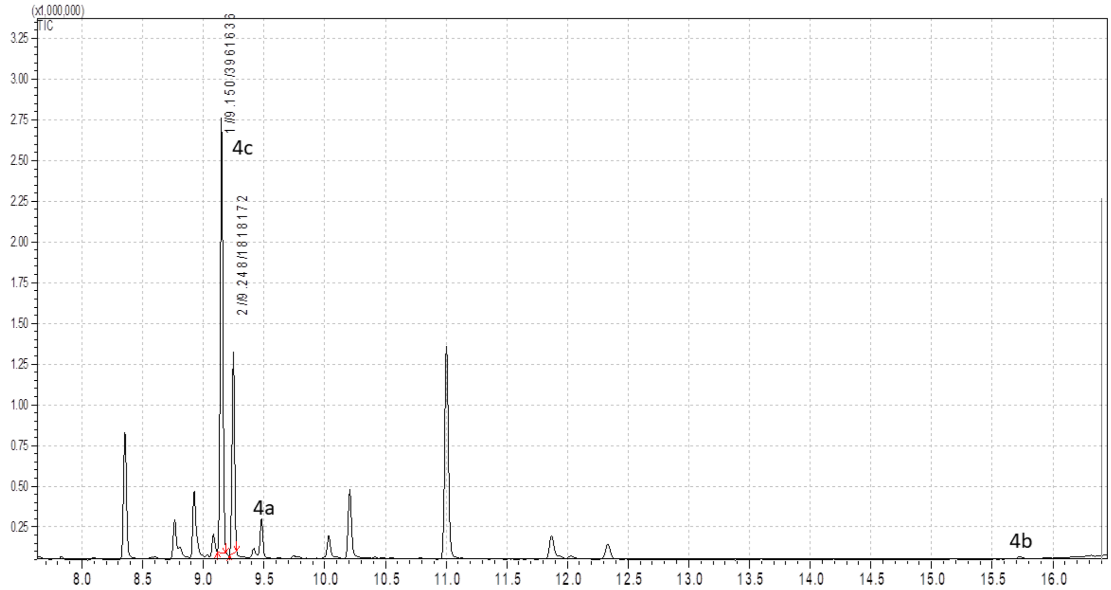

Supplementary Figure 20. Exemplary GC chromatogram of the reaction mixture after silylation (BSTFA) Retention times: **4c**: 9.15 min, **4a**: 9.48 min, **4b**: 15.71 min.

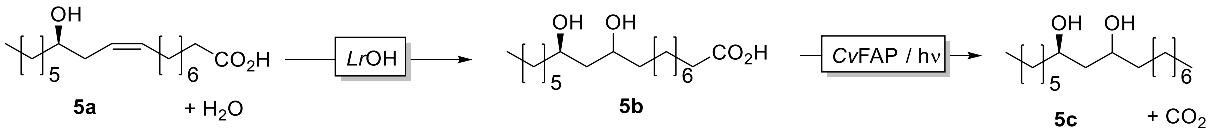

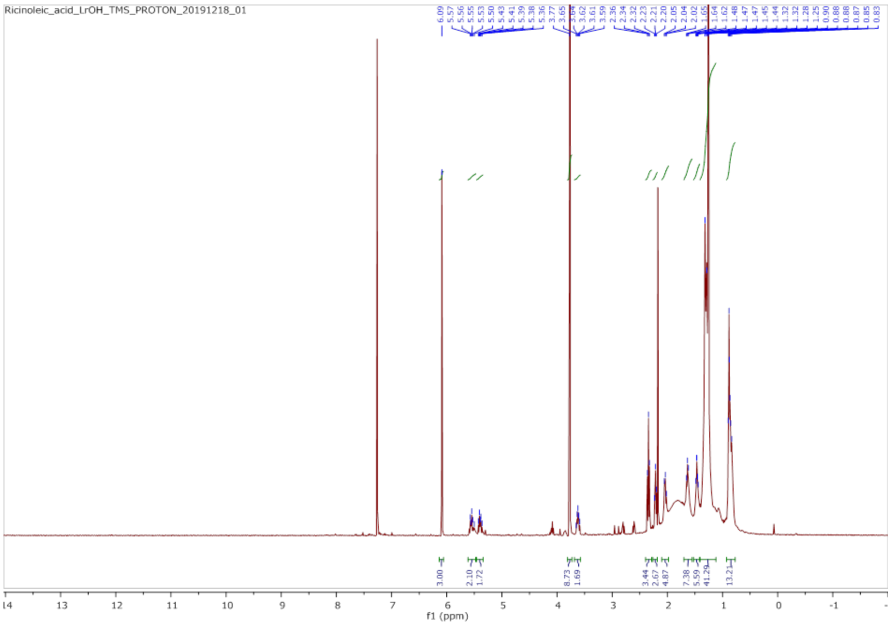

Supplementary Figure 21 ^{1}H NMR spectrum of (12*R*)-10,12-dihydroxyoctadecanoic acid (**5b**).

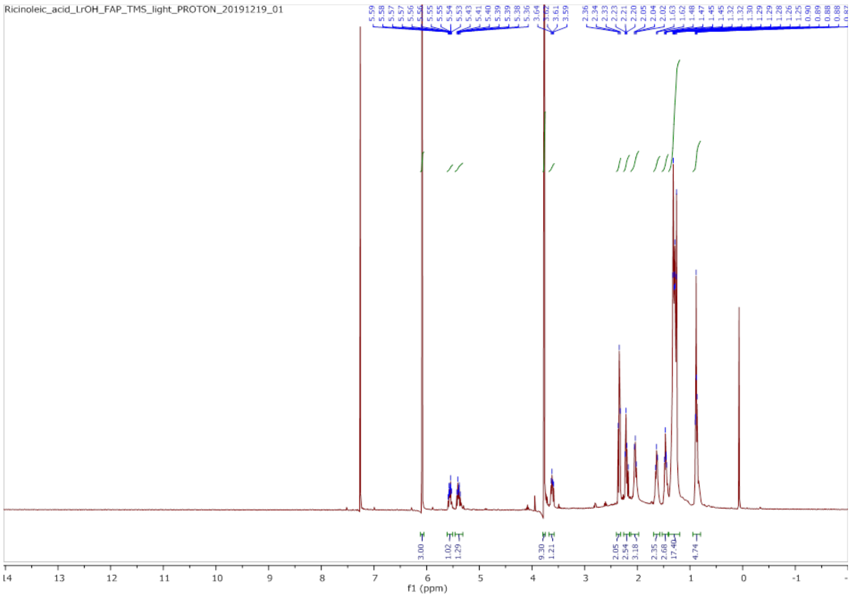

Supplementary Figure 22 ^{1}H NMR spectrum of (7*R*, 9*R*)-heptadecane-7,9-diol (**5c**).

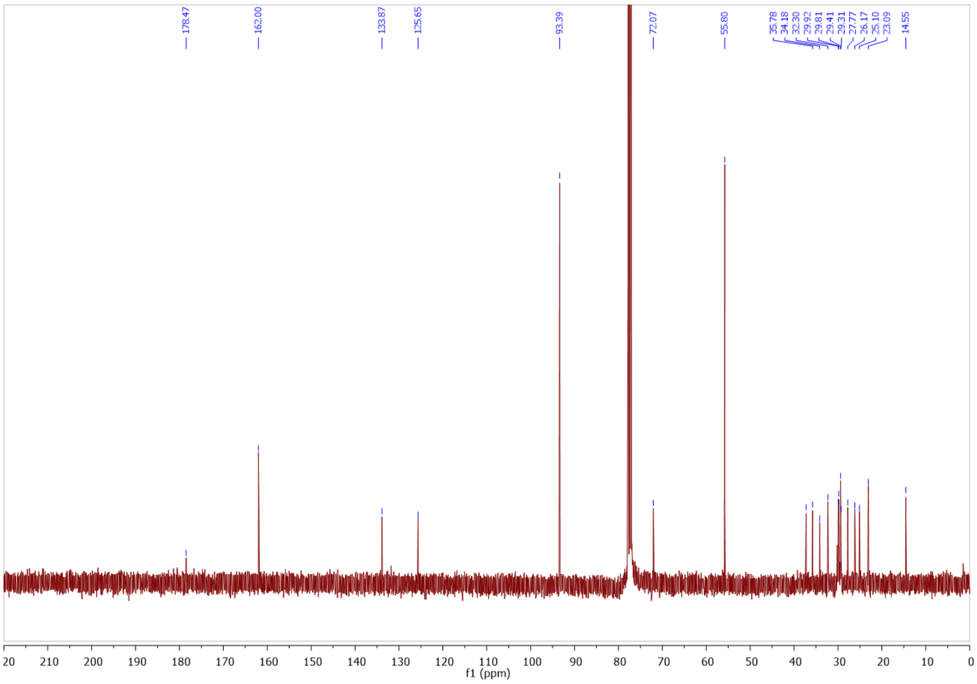

Supplementary Figure 23. ^{13}C NMR spectrum of (7R, 9R)-heptadecane-7,9-diol (**5c**).

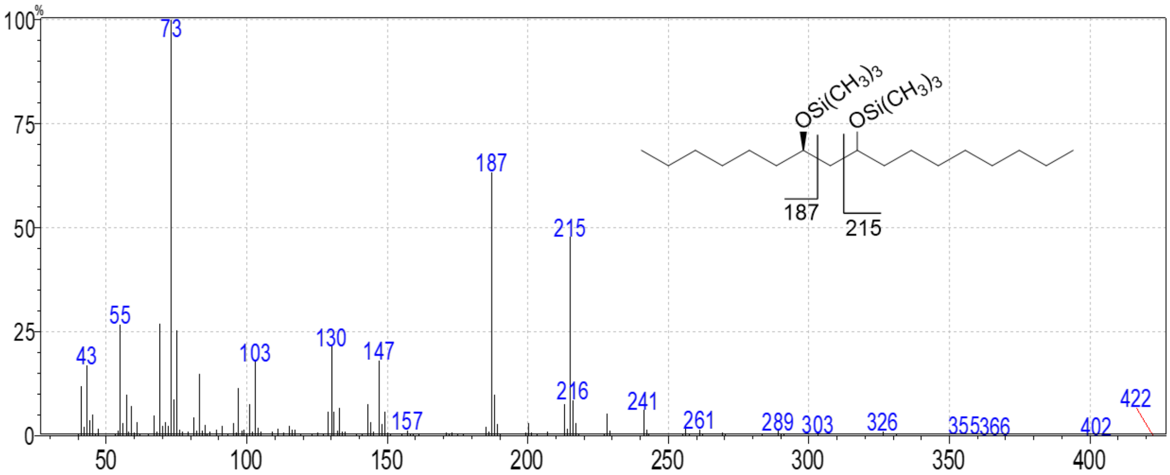

Supplementary Figure 24. GC-MS analysis showing mass spectrum of silylated heptadecane-7,9-diol (**5c**).

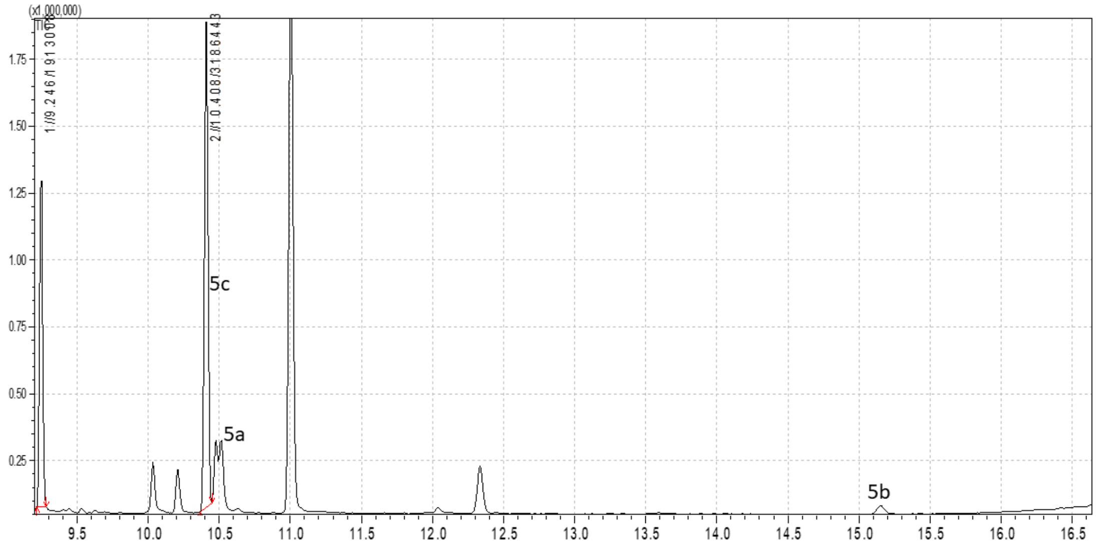

Supplementary Figure 25. Exemplary GC chromatogram of the reaction mixture after silylation (BSTFA). Retention times: **5c**: 10.41 min, **5a**: 10.54min, **5b**: 15.17min.

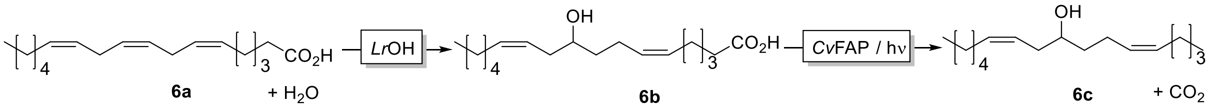

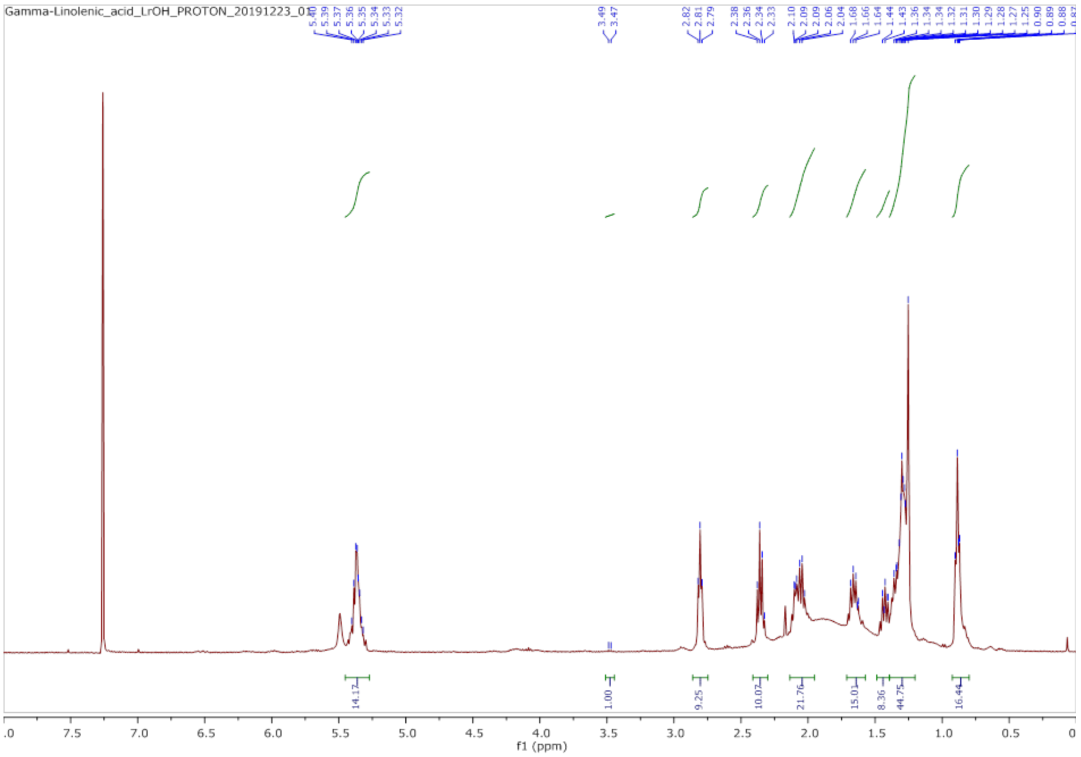

Supplementary Figure 26. ^{1}H NMR spectrum of (*R*,6*Z*,12*Z*)-10-hydroxyoctadeca-6,12-dienoic acid (**6b**).

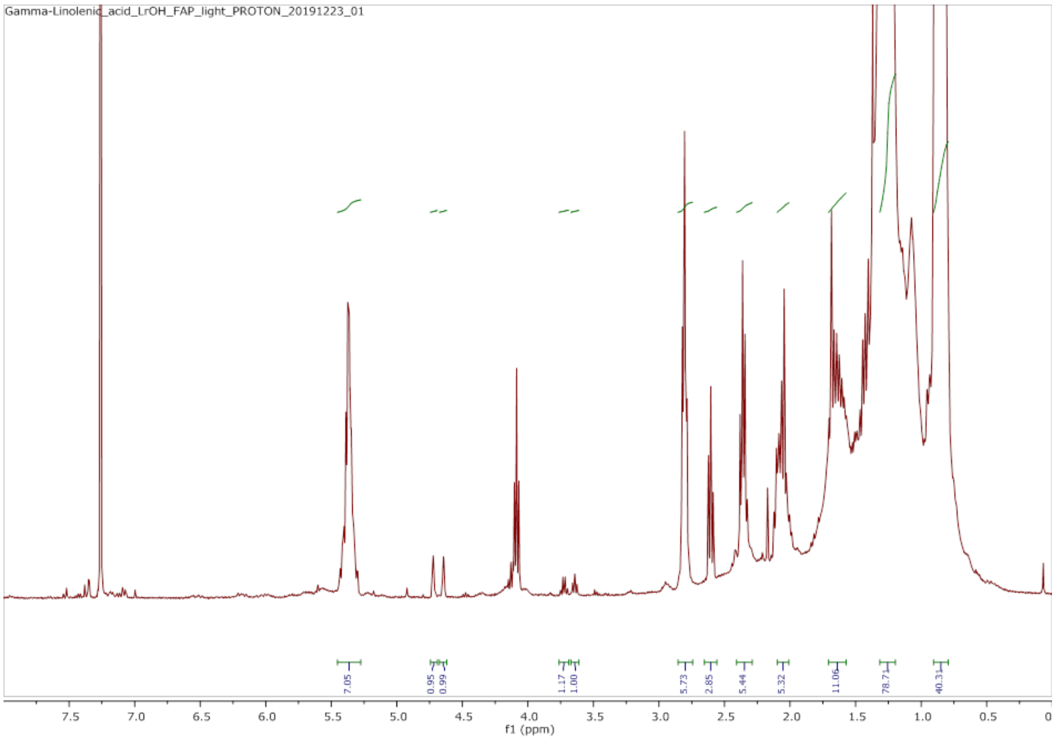

Supplementary Figure 27. ^{1}H NMR spectrum of (*R*,5*Z*,11*Z*)-heptadeca-5,11-dien-9-ol (**6c**).

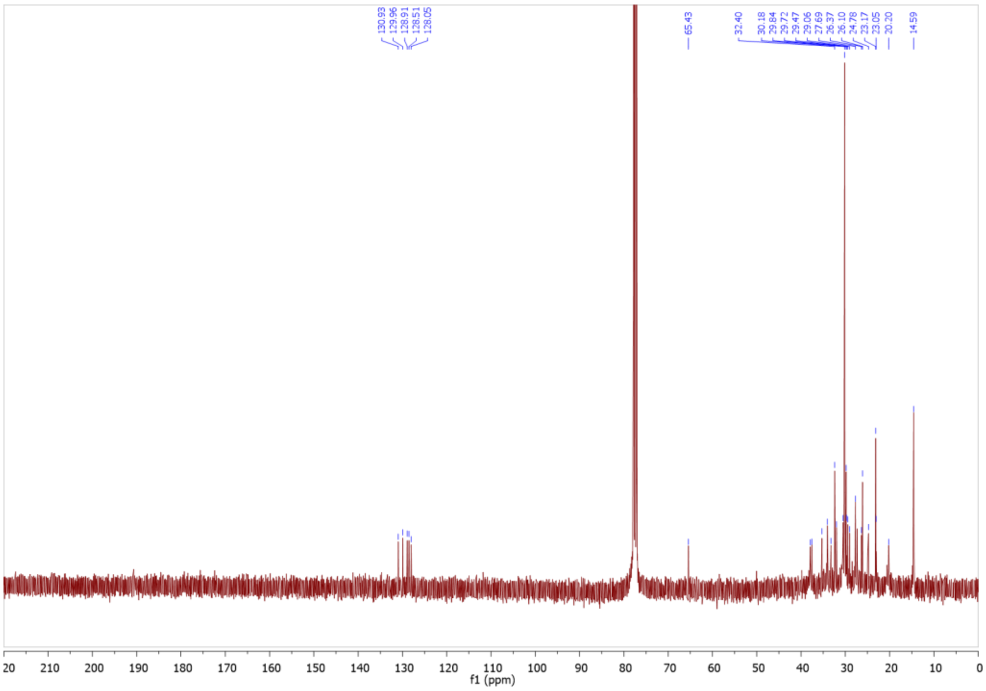

Supplementary Figure 28. ^{13}C NMR spectrum of (R,5Z,11Z)-heptadeca-5,11-dien-9-ol (**6c**).

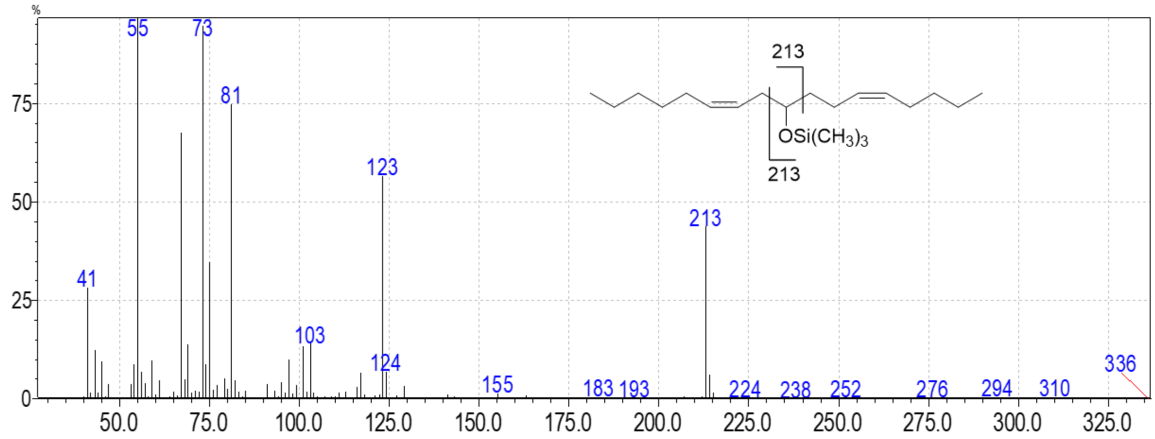

Supplementary Figure 29. GC-MS analysis showing mass spectrum of silylated (5*Z*,11*Z*)-heptadeca-5,11-dien-9-ol (**6c**).

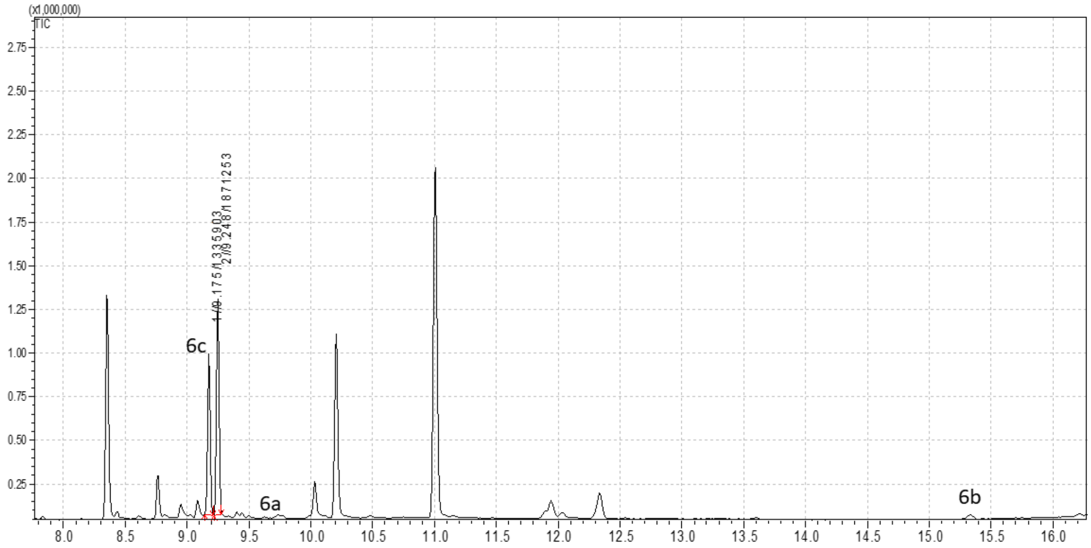

Supplementary Figure 30. Exemplary GC chromatogram of the reaction mixture after silylation (BSTFA). Retention times: **6c**: 9.18 min, **6a**: 9.61 min, **6b**: 15.33 min, 9-heptadecanol 9.25 min.

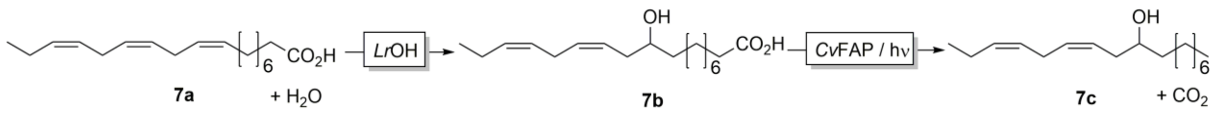

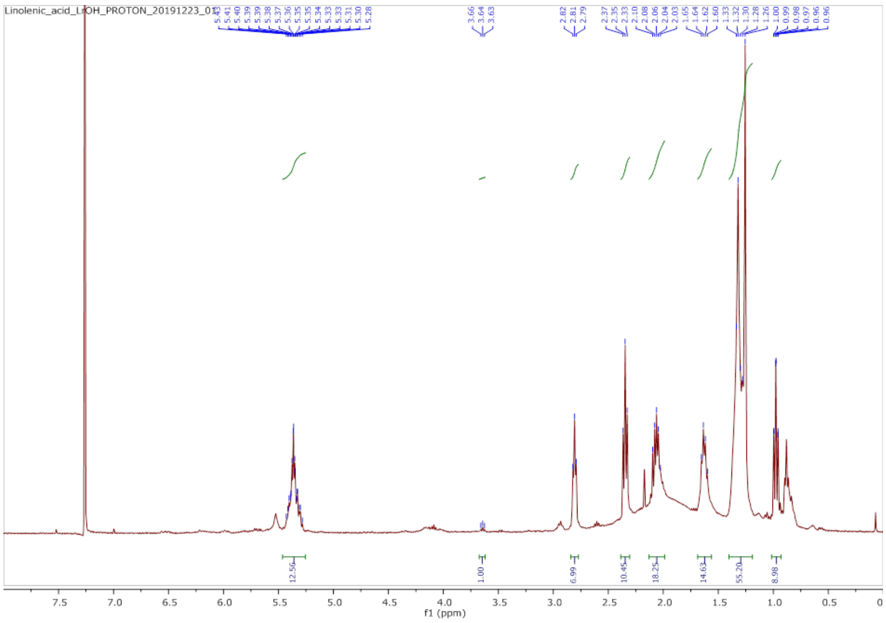

Supplementary Figure 31. ^{1}H NMR spectrum of (*R*,12*Z*,15*Z*)-10-hydroxyoctadeca-12,15-dienoic acid (**7b**).

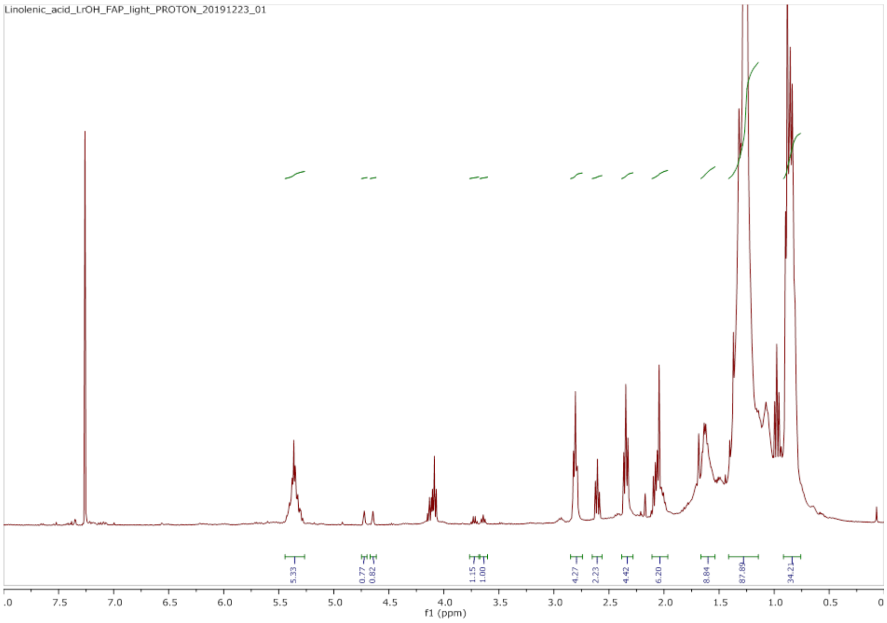

Supplementary Figure 32. ^{1}H NMR spectrum of (*R*,3*Z*,6*Z*)-heptadeca-3,6-dien-9-ol (**7c**).

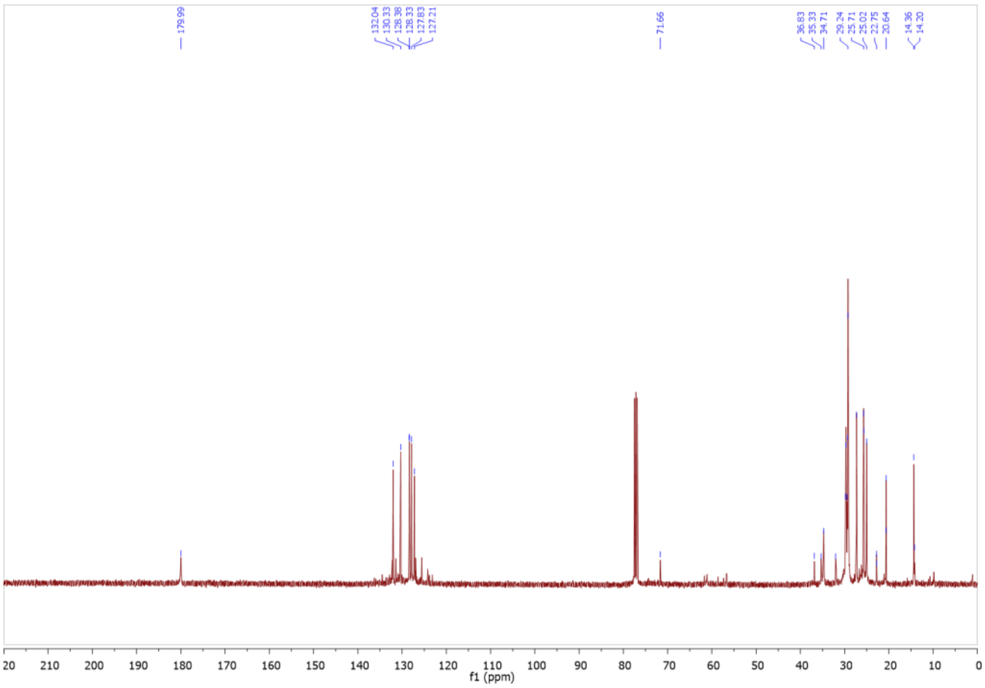

Supplementary Figure 33. ^{13}C NMR spectrum of (R,3Z,6Z)-heptadeca-3,6-dien-9-ol (**7c**).

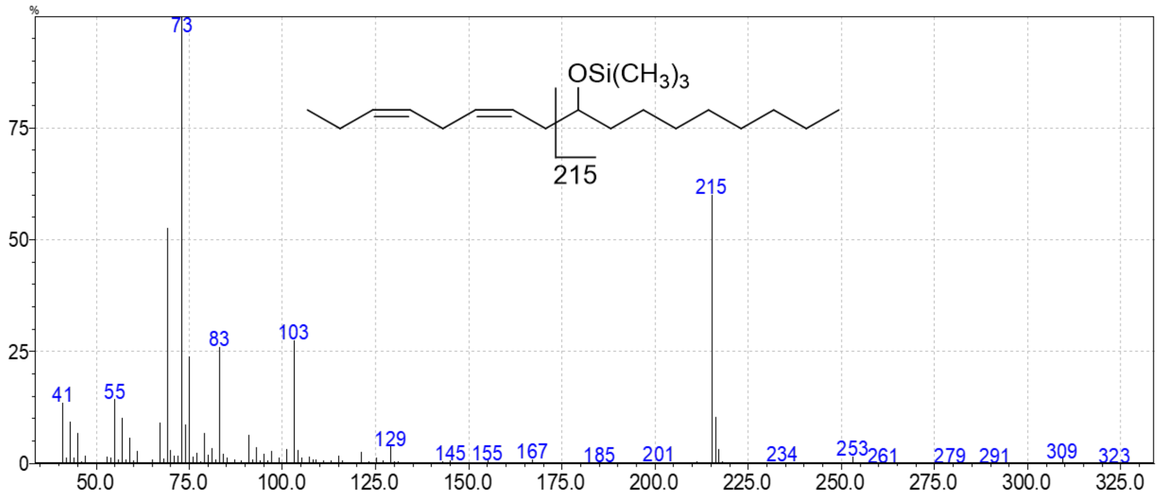

Supplementary Figure 34. GC-MS analysis showing mass spectrum of silylated (3*Z*,6*Z*)-heptadeca-3,6-dien-9-ol (**7c**).

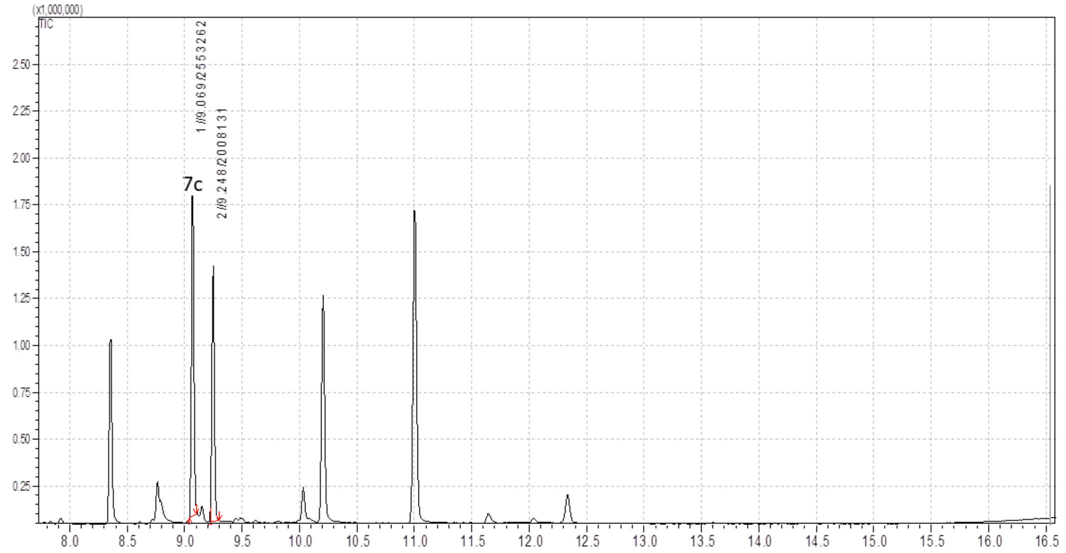

Supplementary Figure 35. Exemplary GC chromatogram of the reaction mixture after silylation (BSTFA). Retention times: **7c**: 9.07 min, 9-heptadecanol 9.25 min.

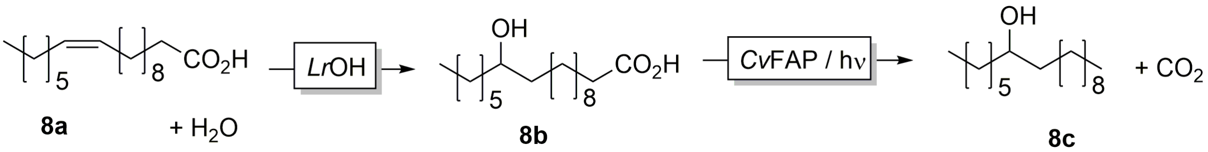

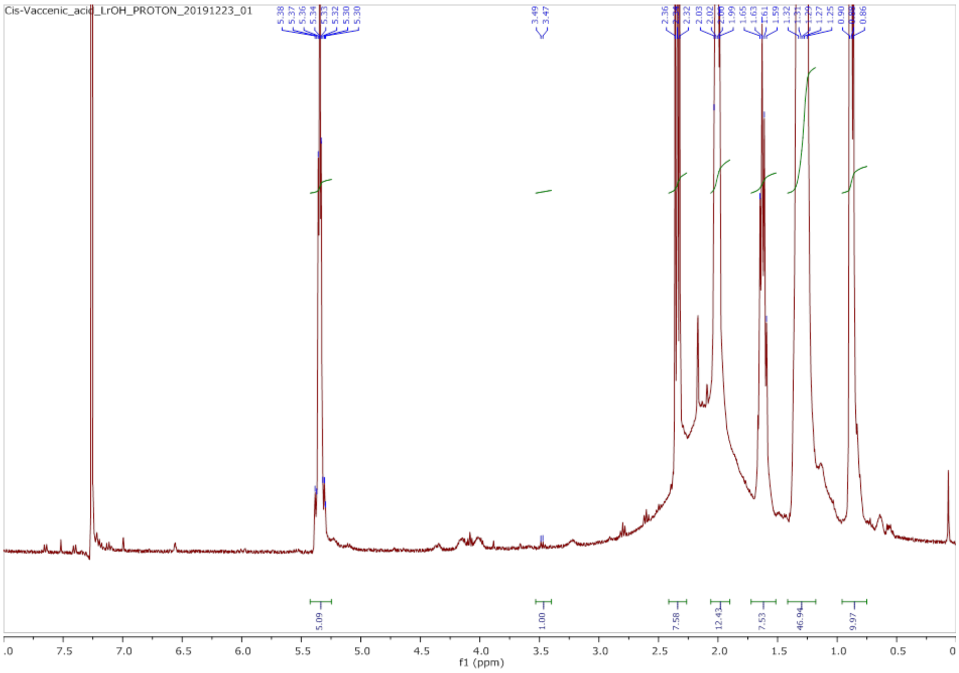

Supplementary Figure 36. ^{1}H NMR spectrum of 12-hydroxyoctadecanoic acid (**8b**).

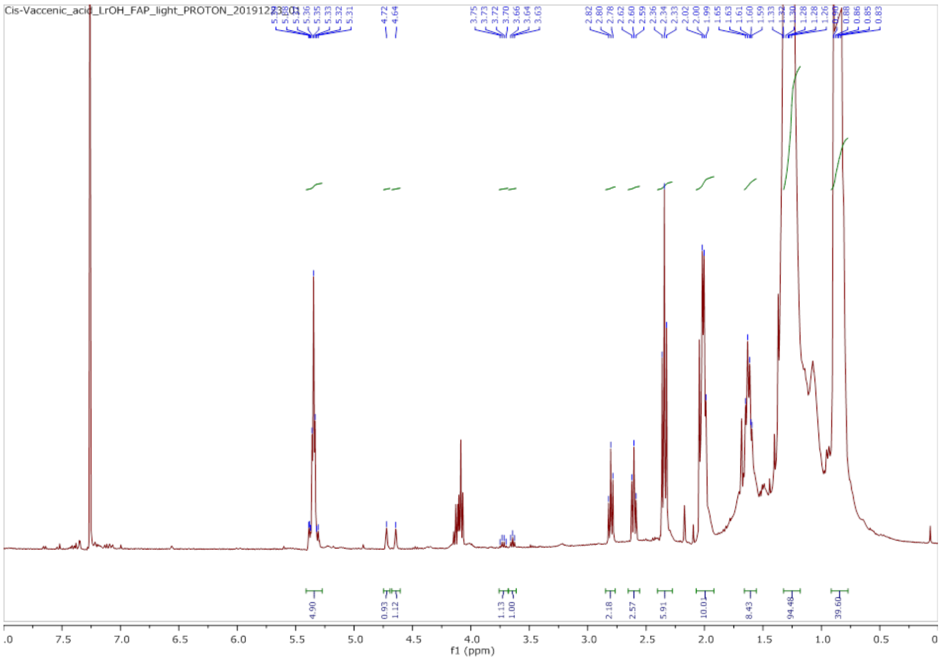

Supplementary Figure 37. ^{1}H NMR spectrum of (*R*)-heptadecan-7-ol (**8c**).

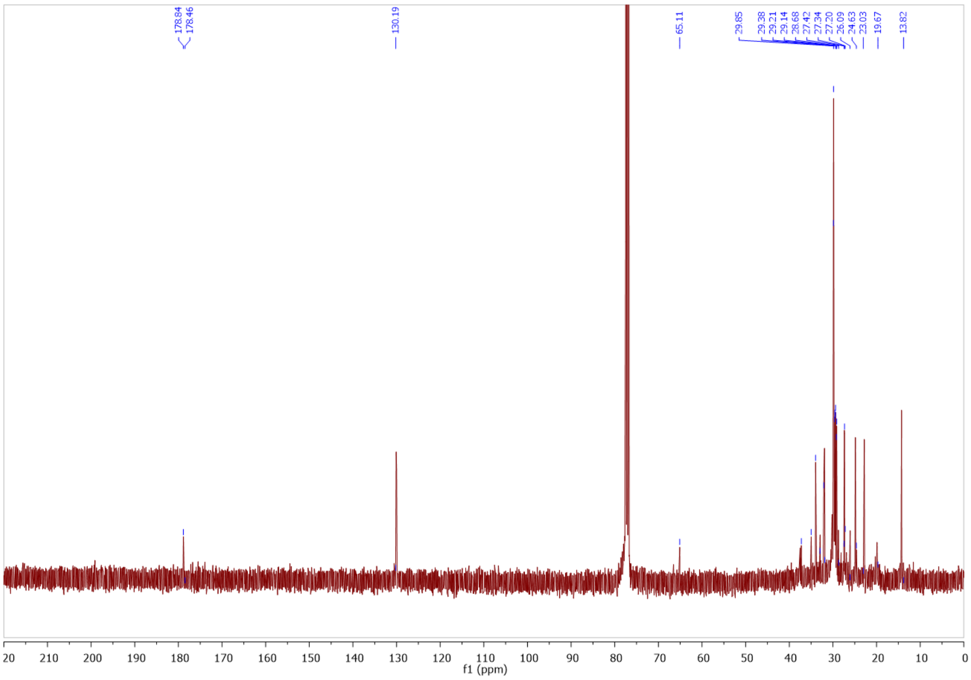

Supplementary Figure 38. ^{13}C NMR spectrum of (R)-heptadecan-7-ol (**8c**).

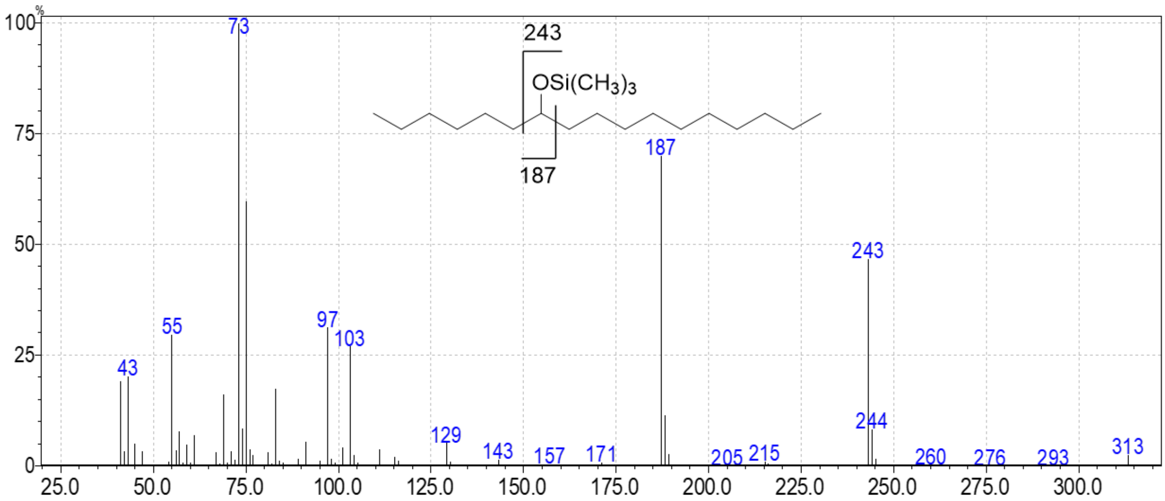

Supplementary Figure 39. GC-MS analysis showing mass spectrum of silylated 7-heptadecanol (**8c**).

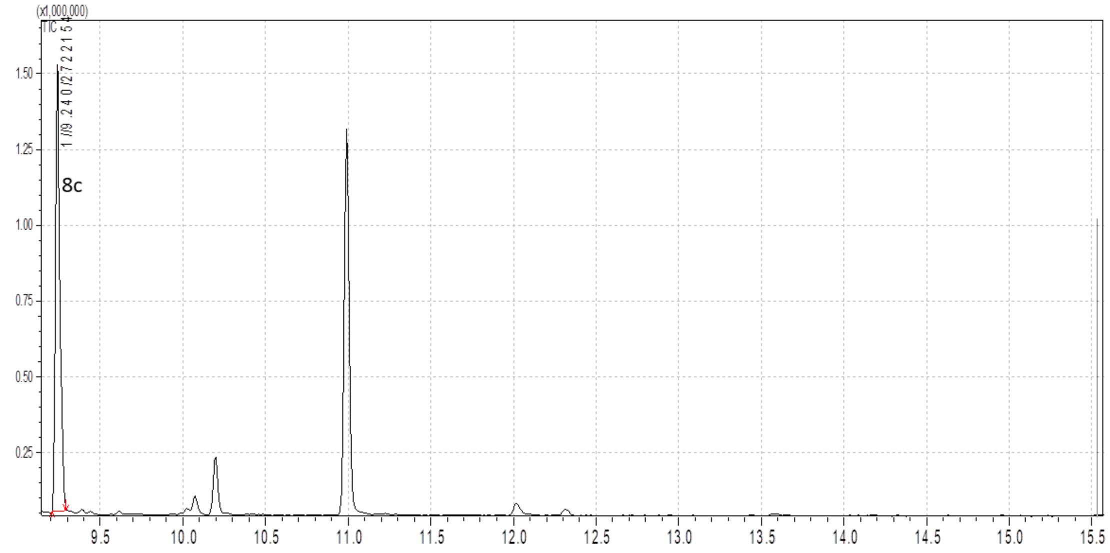

Supplementary Figure 40. Exemplary GC chromatogram of the reaction mixture after silylation (BSTFA). Retention times: **8c**: 9.27 min. All compounds were silylated by using BSTFA.

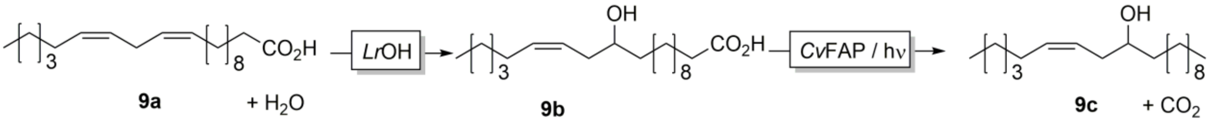

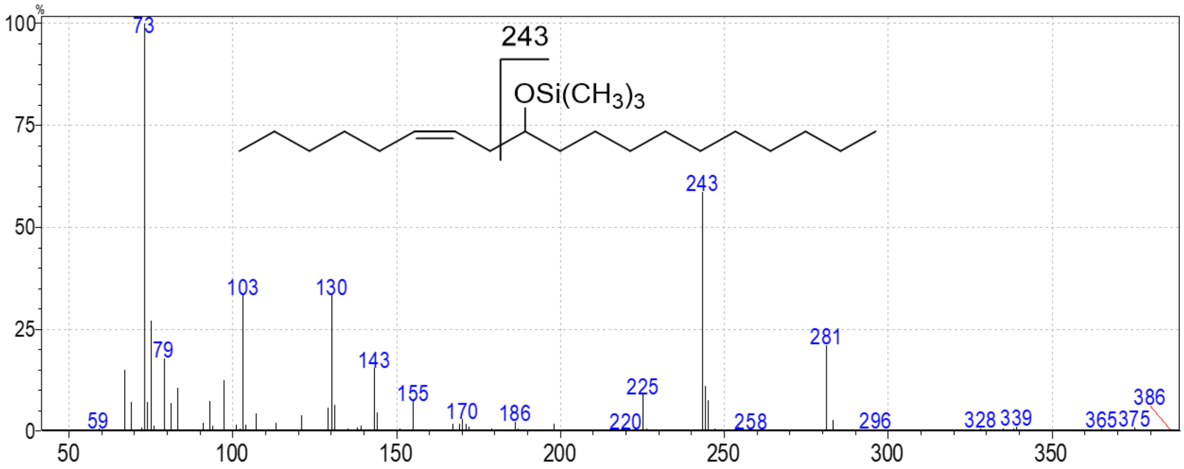

Supplementary Figure 41. GC-MS analysis showing mass spectrum of silylated (*Z*)-nonadec-6-en-9-ol (**9c**).

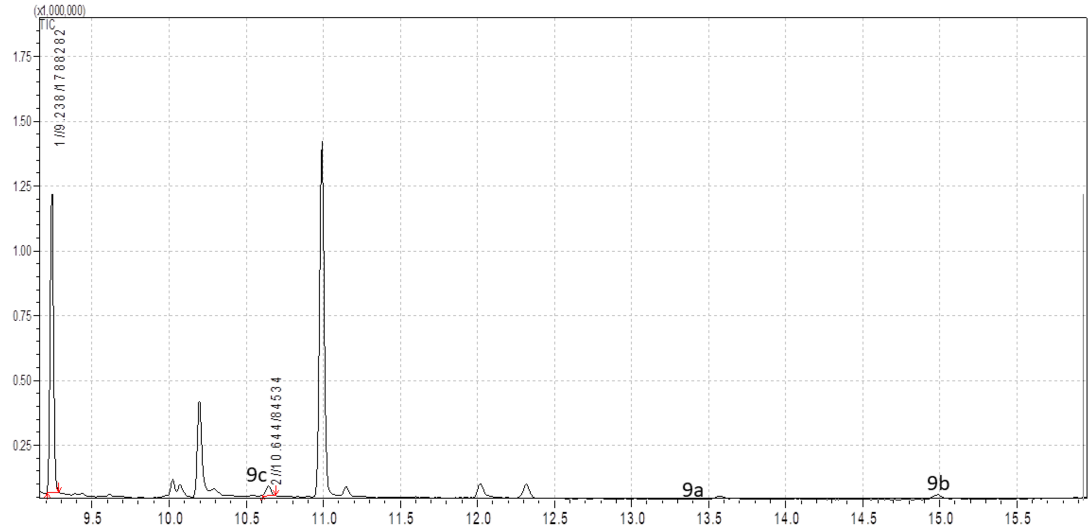

Supplementary Figure 42. Exemplary GC chromatogram of the reaction mixture after silylation (BSTFA). Retention times: **9c**: 10.64min, **9a**: 13.46min, **9b**: 15.10 min, 9-heptadecanol 9.24 min.

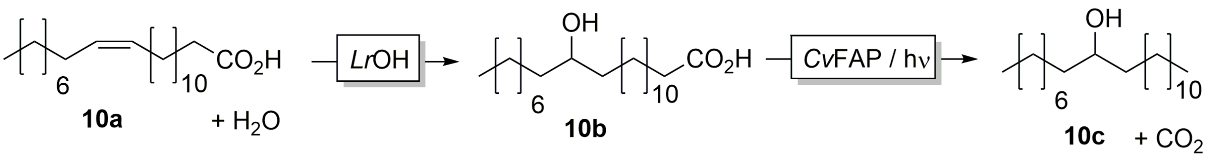

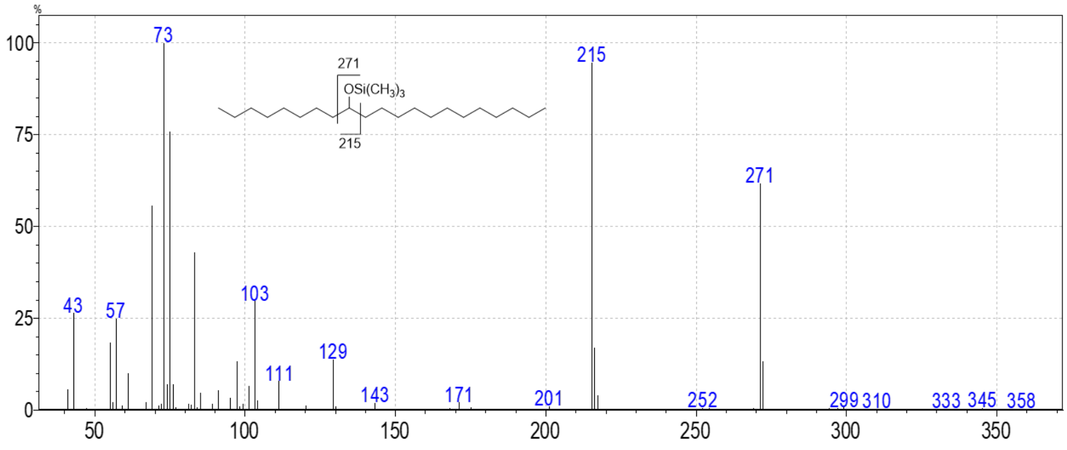

Supplementary Figure 43. GC-MS analysis showing mass spectrum of silylated 9-henicosanol (**10c**).

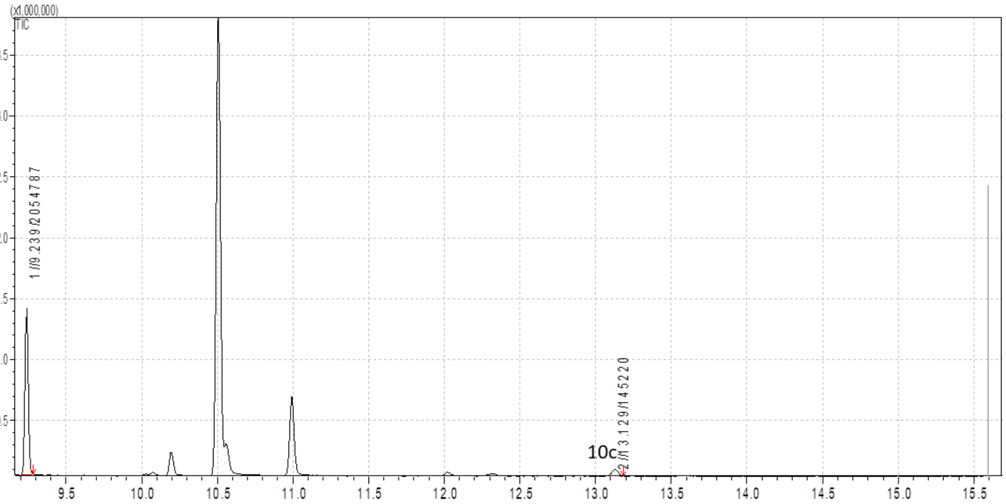

Supplementary Figure 44. Exemplary GC chromatogram of the reaction mixture after silylation (BSTFA). Retention time: **10c**: 13.13min, alkane product ((*Z*)-henicos-9-ene): 10.52 min, 9-heptadecanol 9.24 min.

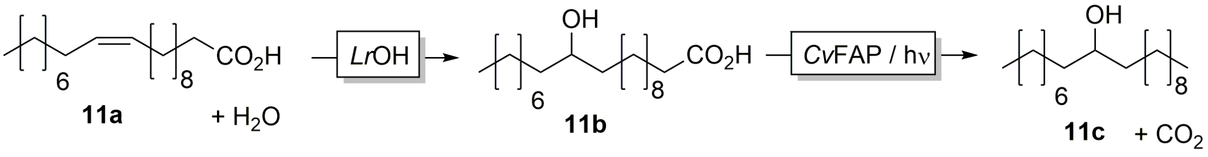

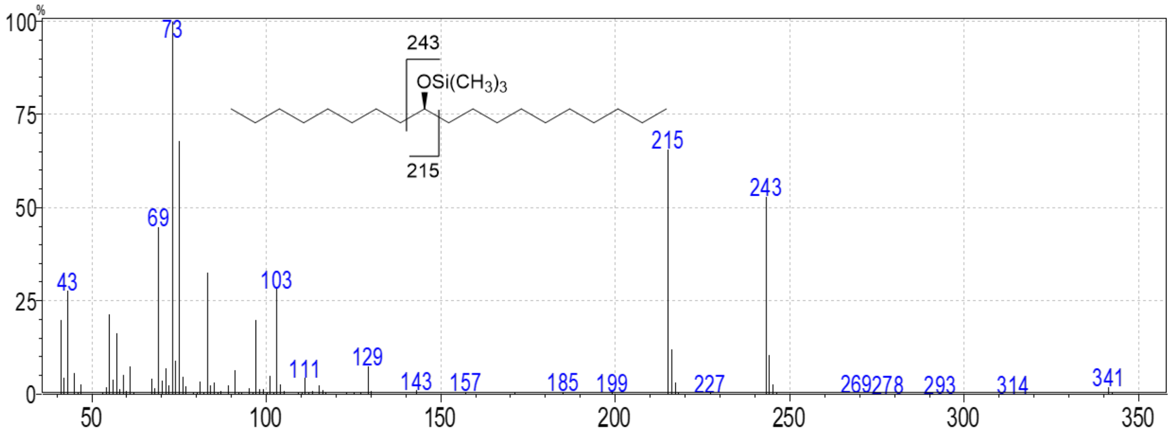

Supplementary Figure 45. GC-MS analysis showing mass spectrum of silylated 9-nonadecanol (**11c**).

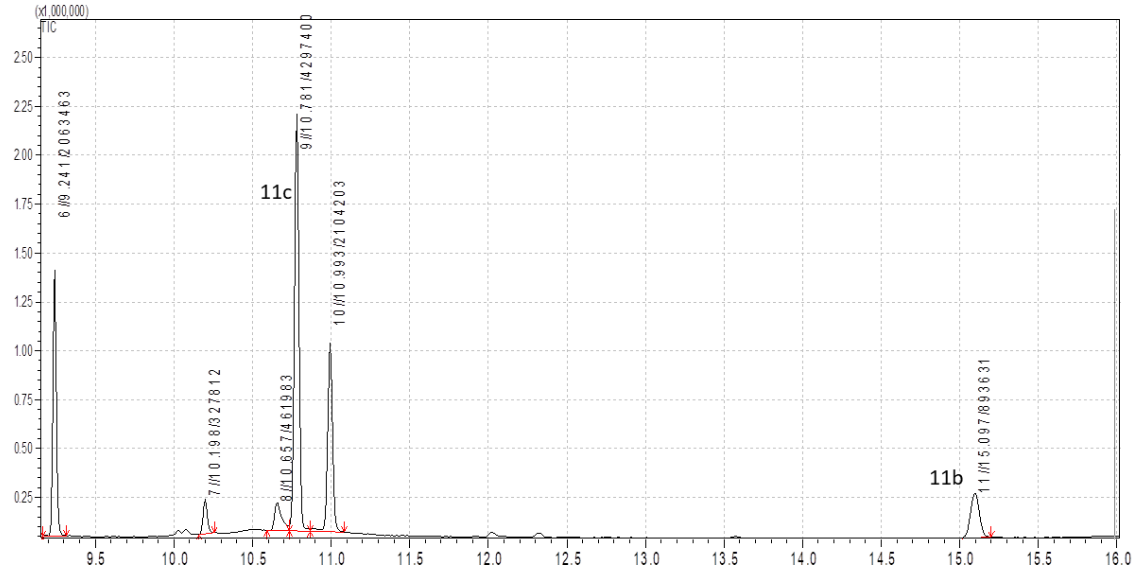

Supplementary Figure 46. Exemplary GC chromatogram of the reaction mixture after silylation (BSTFA). Retention times: **11c**: 10.78min, **11b**: 15.10 min, 9-heptadecanol 9.24 min.

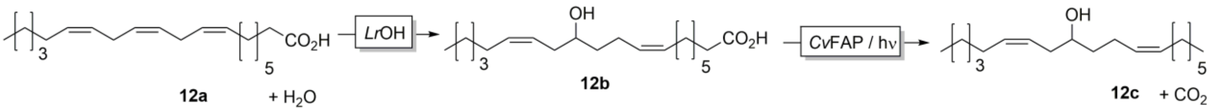

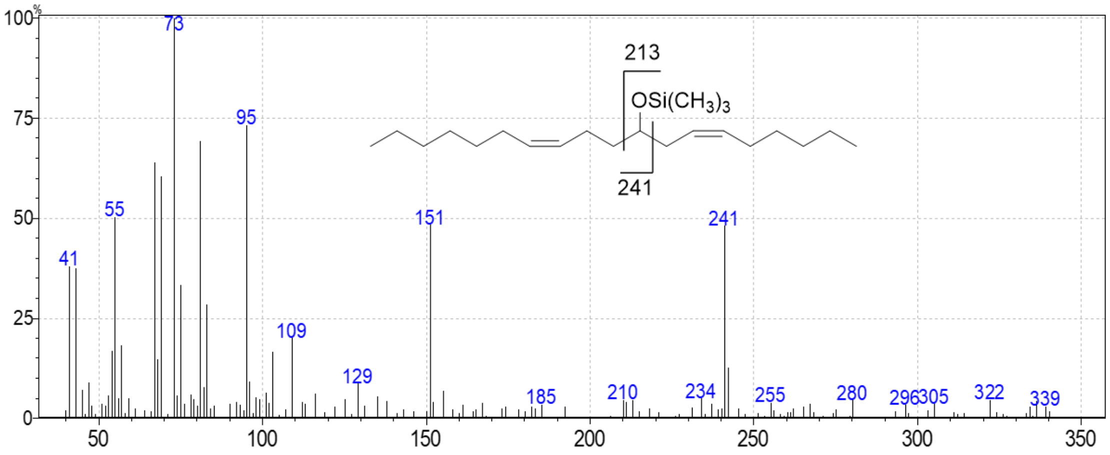

Supplementary Figure 47. GC-MS analysis showing mass spectrum of silylated (6*Z*,12*Z*)-nonadeca-6,12-dien-9-ol (**12c**).

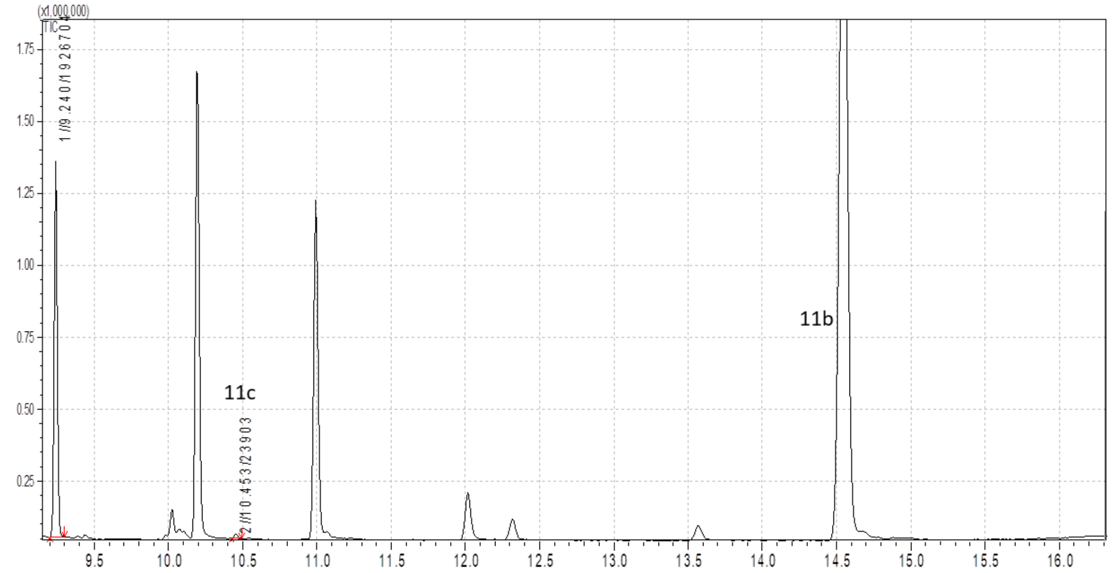

Supplementary Figure 48. Exemplary GC chromatogram of the reaction mixture after silylation (BSTFA). Retention times: **12c**: 10.45 min, **12a**: 14.55 min, 9-heptadecanol 9.24 min.

2c

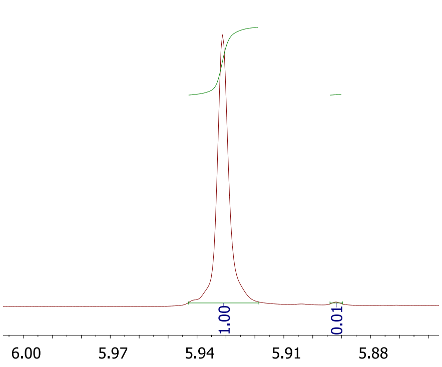

3c

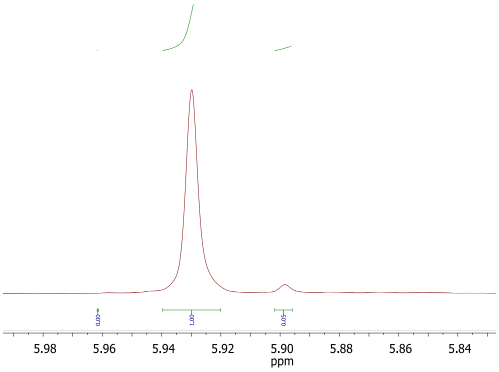

4c

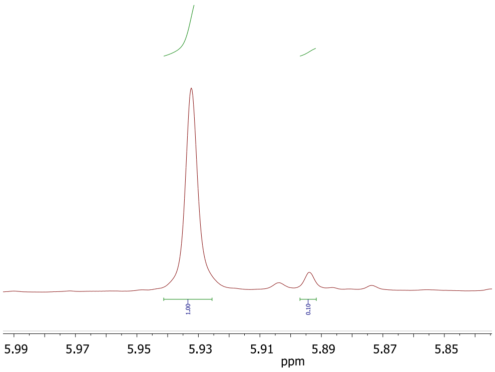

5c

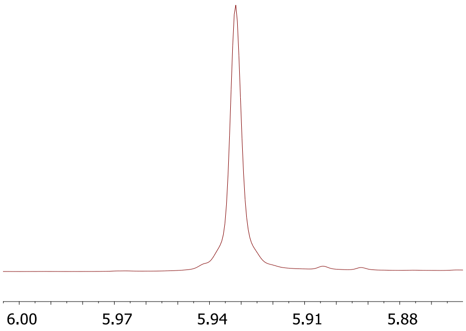

6c

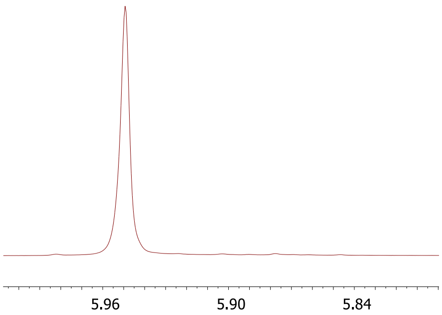

7c

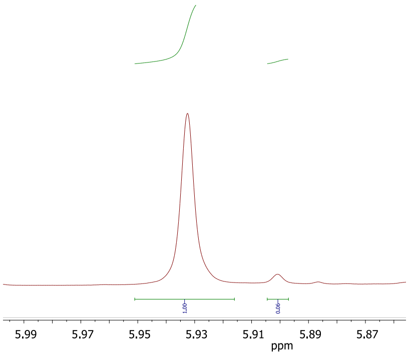

8c

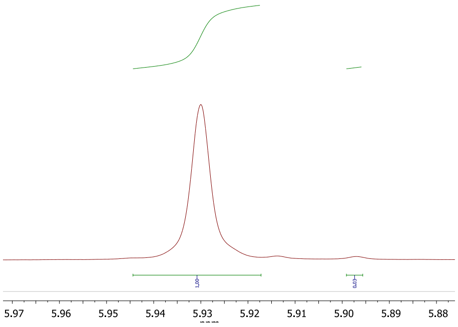

11c

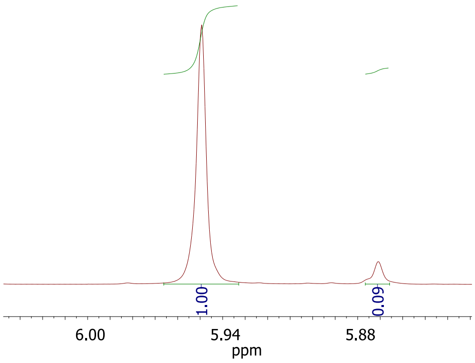

Supplementary Figure 49. ^{1}H NMR spectra ($CDCl_3$, 400 MHz) for determining the optical purity of the alcohol products. The products were derivatised by (*S*)-(+)-*O*-acetylmandelic acid.[1]

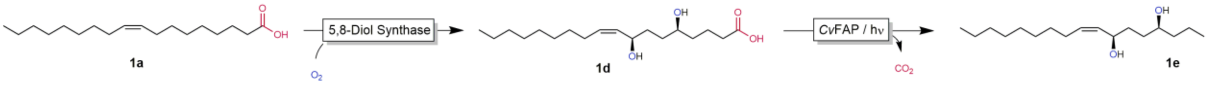

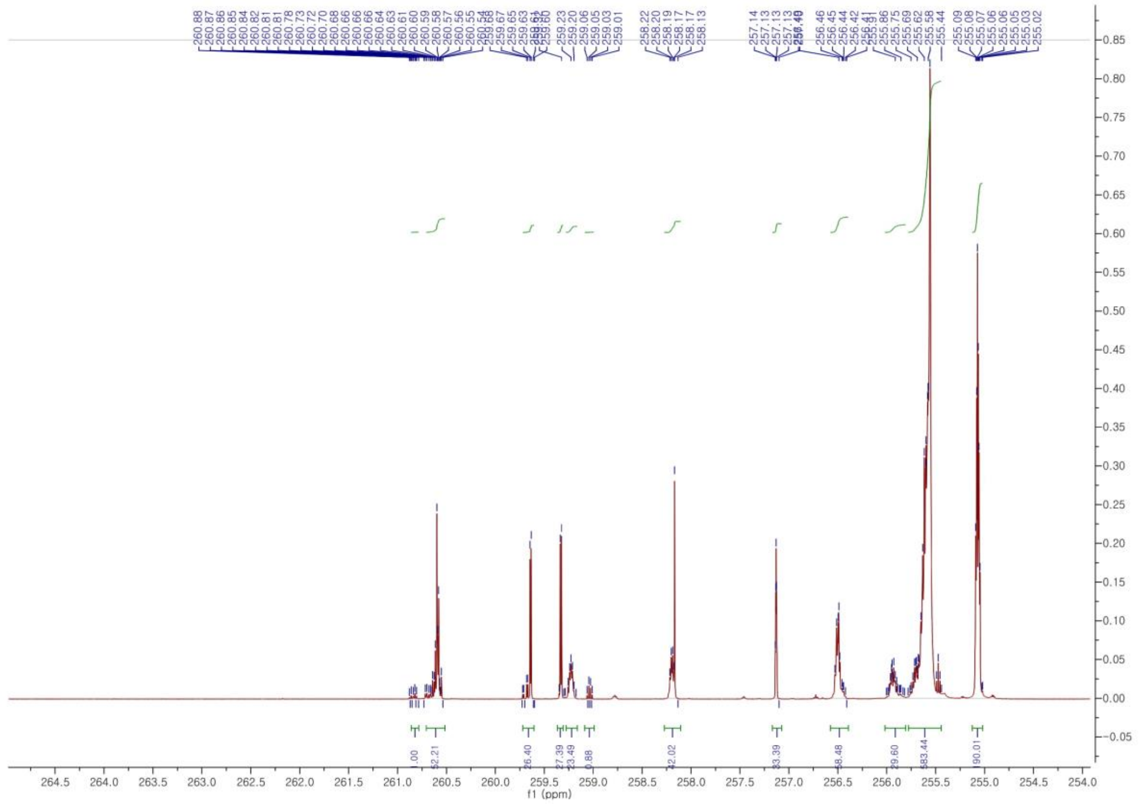

Supplementary Figure 50. ^{1}H NMR spectrum of (4*S*,7*R*,*Z*)-heptadec-8-ene-4,7-diol (**1e**). It is the product from the starting substrate oleic acid. ^{1}H NMR (300 MHz, DMSO-d_6) δ 5.30–5.23 (m, 2H), 4.50 (d, *J* = 3.0 Hz, 1H), 4.25 (d, *J* = 3.0 Hz, 1H), 4.18–4.16 (m, 1H), 3.36–3.32 (m, 1H), 1.99–1.97 (m, 2H), 1.55–1.23 (m, 20H), 0.86–0.80 (m, 6H).

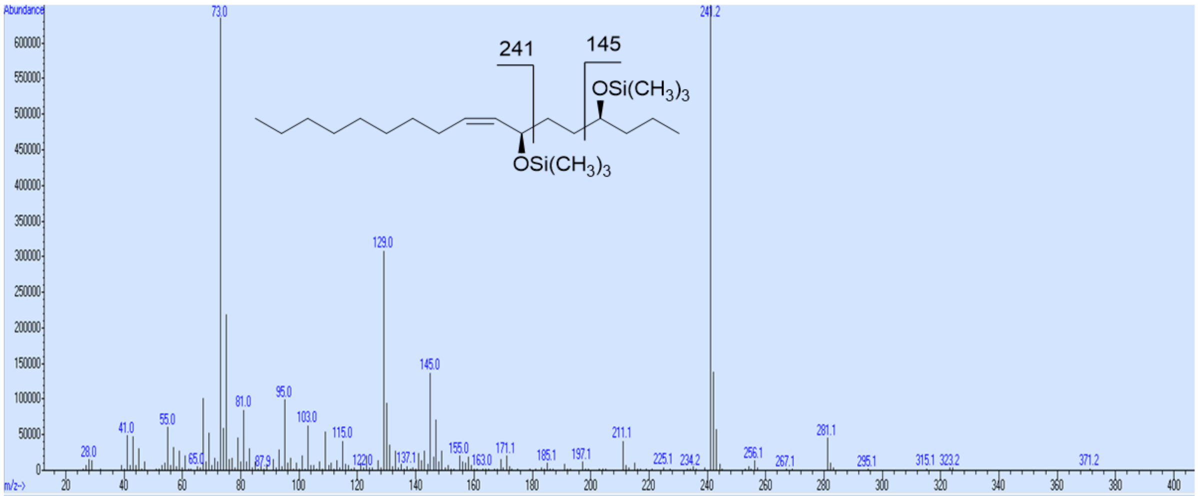

Supplementary Figure 51. GC-MS analysis showing mass spectrum of TMS-derivatised **1e**.

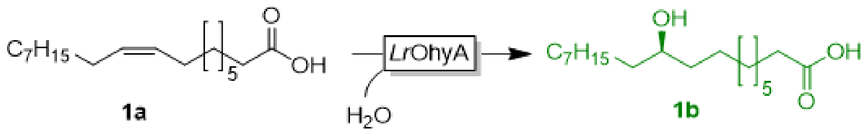

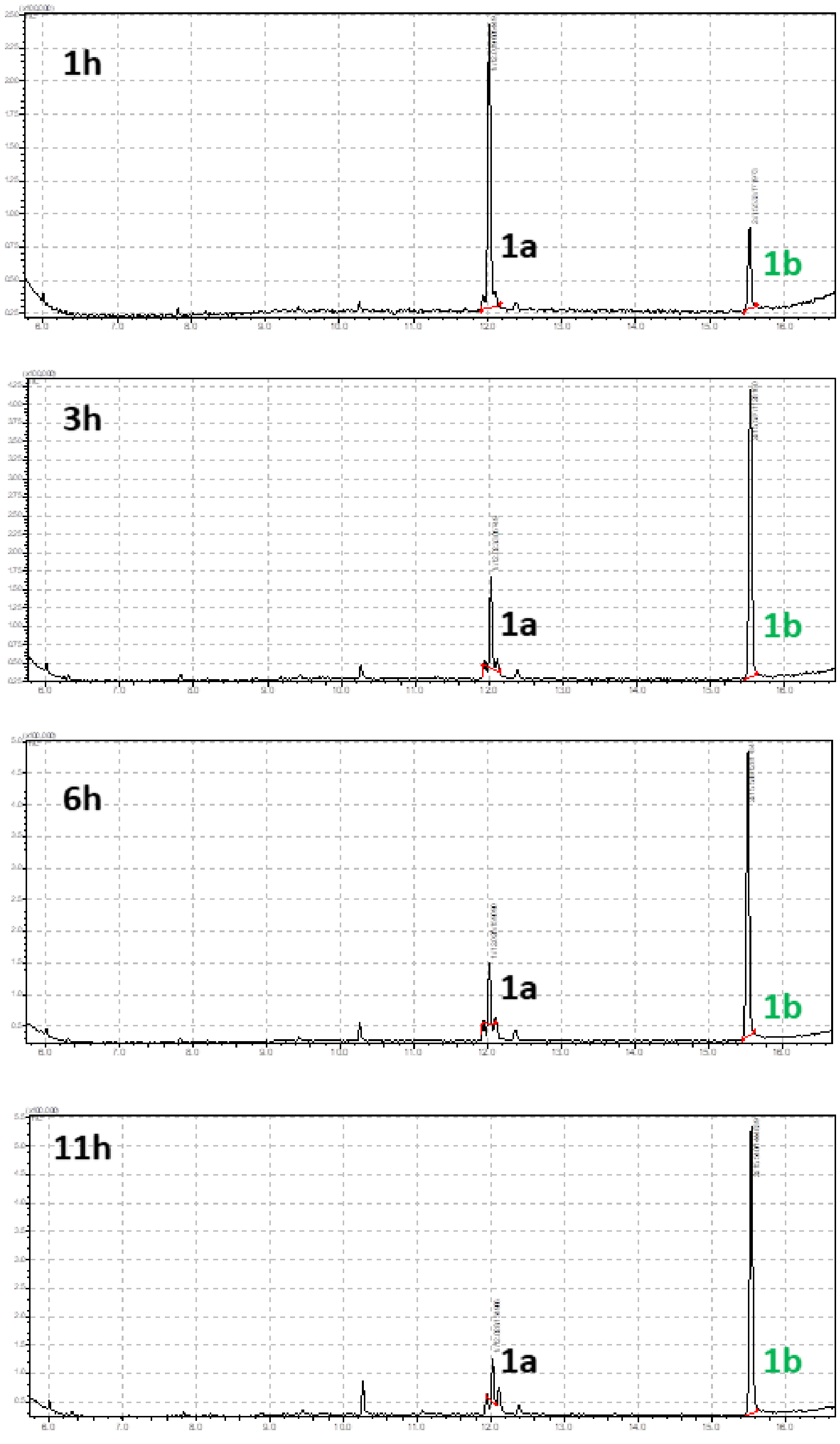

Supplementary Figure 52. Exemplary GC chromatograms of the time course shown in Figure 3.

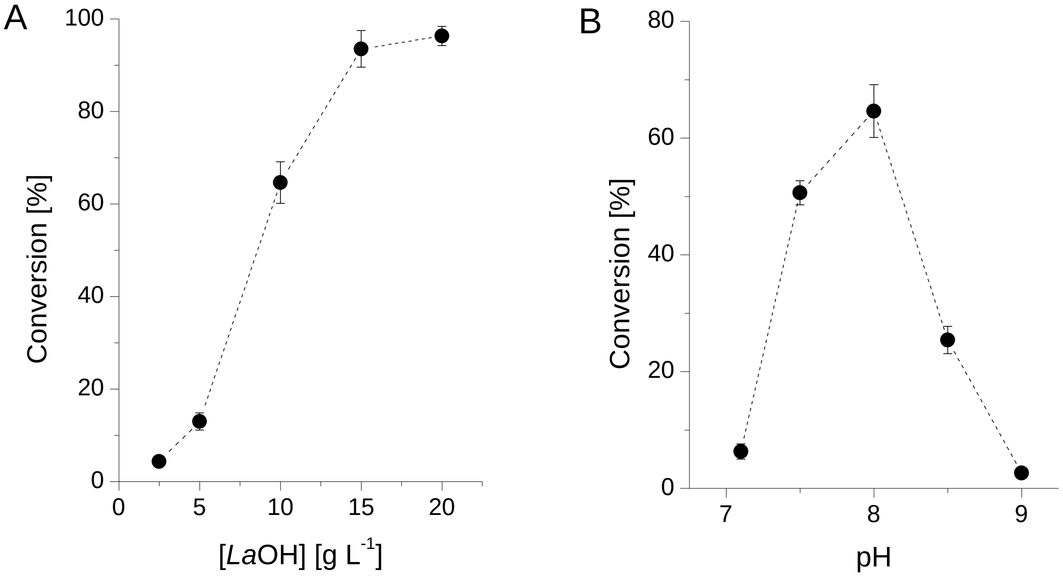

Supplementary Figure 53. Photoenzymatic hydratation-decarboxylation of oleic acid. (A) Influence of the *Lr*OH concentration, [*Lr*OH@*E. coli*] = 2.5-20 gL^{-1} at pH 8.0 and (B) influence of the reaction pH, [*Lr*OH@*E. coli*] = 10 gL^{-1}. For all the reactions, first the *Lr*OH-catalysed hydratation reaction was performed for 11h followed by addition of *Cv*FAP and illumination for another 6 h at 30 °C. Values represent the average of duplicates (n=2). Error bars indicate the standard deviation

A

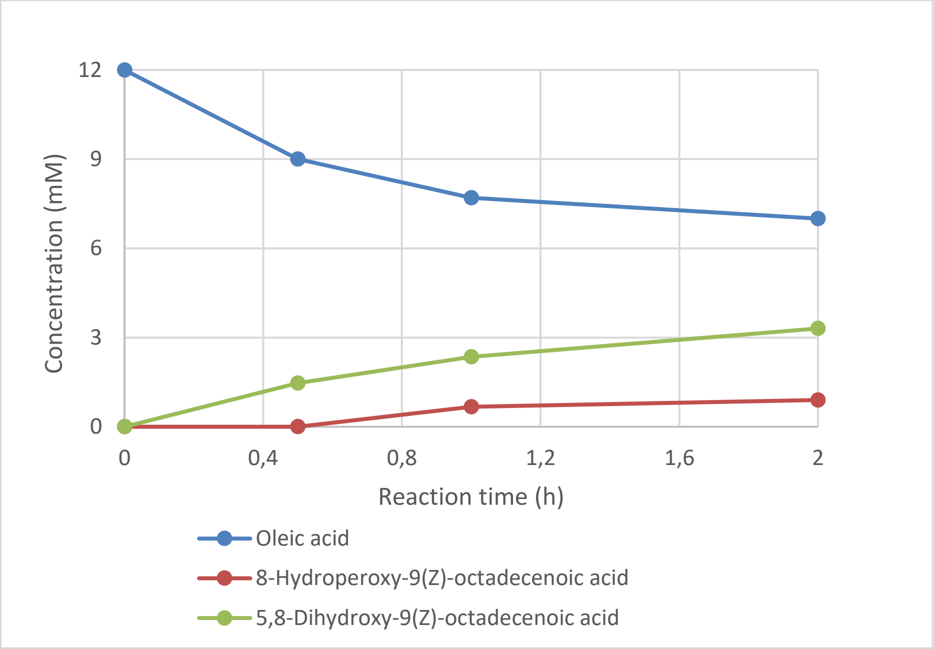

B

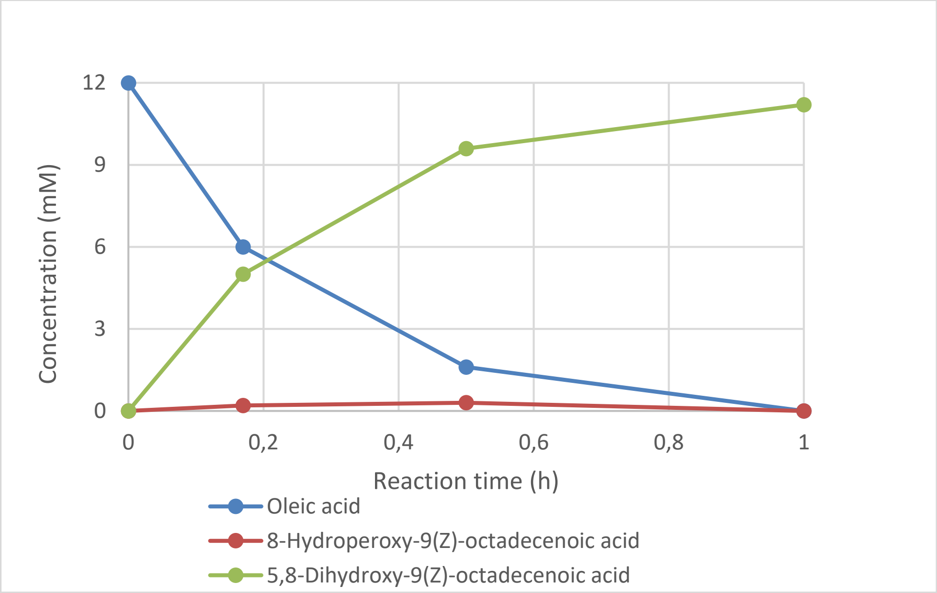

Supplementary Figure 54. Exemplary time course for the transformation shown in Figure 7. Biotransformation of oleic acid into 5,8-dihydroxy-9(*Z*)-octadecenoic acid by (A) *E. coli* BL21(DE3) pET21a-*An*DS and (B) *E. coli* BL21(DE3) pACYC-PelBSS-*An*DS. Typical time course [oleic acid] = 12 mM, [*An*DS @*E. coli*] = 7 gL^{-1}, HEPS buffer pH 7.5 (50mM, with 10 % (v/v) DMSO): oleic acid (■), 8-hydroperoxy-9(Z)-octadecenoic acid (8-HPOME) (○), 5,8-dihydroxy-9(*Z*)-octadecenoic acid (5,8-diHOME) (●).

Supplementary Table 1. Quantification of the conversion of hydroxyl fatty acids using ^{1}H NMR.

Entry	Product	Product conc. mM	Conversion, %
1	3b	2.9	29
2	4b	2.6[a]	52
3	5b	8	80
4	6b	4.1	41
5	7b	4.4	44
6	8b	6.6	66

^{1}H NMR was used by integrating the characteristic peaks of the substrate (5.35 ppm from _H_C=C_H_) and the product (C_H_-OH), duplicate experiments were performed. Reaction conditions: [substrate] = 10 mM, [*Lr*OhyA-cells] = 20 gL^{-1}, Tris-HCl buffer (100 mM, with 50 mM of NaCl). The reactions were performed for 11 h. a: [substrate] = 5 mM.

Supplementary Table 2. Quantification of the overall conversion using ^{1}H NMR.

Entry	Product	Product conc. mM	Conversion, %
1	3c	3.1	31
2	4c	5.7	65
3	5c	6	75
4	6c	4.4	43
5	7c	4.2	44
6	8c	4.6	46

^{1}H NMR was used by integrating the characteristic peaks of the substrate (5.35 ppm from _H_C=C_H_) and the product (C_H_-OH), duplicate experiments were performed. Reaction conditions: [substrate] = 10 mM, [*Lr*OhyA-cells] = 20 gL^{-1}, [*Cv*FAP] = 2 µM, Tris-HCl buffer (100 mM, with 50 mM of NaCl), blue light (λ = 450 nm; intensity=13.7 mEL^{-1}s^{-1}). The reactions were performed in a two-step fashion: first the LrOhyA -catalysed hydratation reaction was performed for 11 h followed by addition of *Cv*FAP and illumination for another 6 h.

Supplementary Table 3. Product formation in the biphasic cascade reactions.

Time, h	Oleic acid, mM	*(R)*-10-hydroxystearic acid, mM	9-heptadecanol, mM
0	20.13	0	n.d.
7	6.36	14.22	n.d.
24	1.85	16.84	n.d.
Addition of *Cv*FAP and switching on the LED			
3.5	n.d.	7.55	9.06
15	n.d.	0.63	17.38

Reaction condition: [oleic acid in triolein] = 20 mM, [*E. coli* overexpressing *Lr*OH] = 10 gL^{-1}, [*Cv*FAP] = 2 µM, Tris-HCl buffer pH 8.0 (100mM, with 50 mM of NaCl), illumination with blue light (λ = 450 nm; intensity = 13.7 mEL^{-1}s^{-1}), triolein : Tris-HCl buffer = 1 :1 , 1.0 mL scale. For the reactions, first the *Lr*OH-catalysed hydratation reaction was performed for 24h followed by addition of *Cv*FAP and illumination for another 15 h at 30 °C.

Supplementary Methods

Chemicals. Unless indicated otherwise all chemicals were purchased from Sigma-Aldrich in the highest purity available and used without further treatment. 9-heptadecanol was purchased from TCI (in Belgium). *(R)*-10-hydroxystearic acid was a gift from InnoSyn B.V. (The Netherlands). Lipase from *Candida rugosa* (890 units/mg) was bought from Aldrich (in 1989) and used directly.

Preparation of the photodecarboxylase *Cv*FAP from *Chlorella variabilis* NC64A

For the production of the fatty acid photodecarboxylase (*Cv*FAP) with short-length in *E. coli*, the procedures were adopted from our previous study.[1, 2] Typically, 10 mL pre-cultures were inoculated with *E. coli* BL21 (DE3) cells harboring the designed pET28a-His-TrxA-*Cv*FAP plasmid. These cultures were grown overnight in terrific broth (TB) medium, containing 50 µg/mL kanamycin. The pre-cultures were used to inoculate large cultures (500 mL TB + 50 µg/mL kanamycin in 2 L shake flasks). Cells were grown at 37 °C, 180 rpm, until an OD_{600} between 0.7-0.8 was reached. Protein production was induced by the addition of 0.5 mM isopropyl-β-D-thiogalactopyranoside (IPTG) and the cells were left at 17 °C, 180 rpm, for about 20 hours. Cells were harvested by centrifugation (11000 g at 4 °C for 10 min) and resuspended directly into the *An*DS reaction medium. Otherwise, the cells, which were harvested (centrifugation at 11000 g, 4 °C for 10 min), were washed with Tris-HCl buffer (50 mM, pH 8, 100 mM NaCl) and centrifuged again. The cell pellet was resuspended in the same buffer, and 1 mM PMSF was added. Cells were lysed by passing them passed twice through a Multi Shot Cell Disruption System (Constant Systems Ltd, Daventry, UK) at 1.5 bar, followed by centrifugation of the cell lysate (38000 g at 4 °C for 1 h). After centrifugation, 5% glycerol (w/v) was added to the soluble fraction, the cell extract was aliquoted, frozen in liquid nitrogen and stored at -80 °C.

The total protein content of the cell extract was determined by a BCA Assay (Interchim), using BSA as a standard. *Cv*FAP production was analysed by SDS-PAGE using a Criterion™ Cell electrophoresis system (Bio-Rad).[2] As a molecular weight marker, Precision Plus Protein Standard (Bio-Rad) was used. The gel was analysed using a gel imaging system (GBox, Syngene, Cambridge, UK) and the amount of *Cv*FAP in the cell extract was estimated from the relative intensity of the bands on gel.

As a control, a cell free extract of *E. coli* BL21 (DE3) cells harboring an empty pET28a vector was prepared according to the same protocol.

Preparation of the oleate hydratase *Lr*OH from *Lactobacillus reuteri*

For the production of the oleate hydratase (*Lr*OH) in *E. coli*, the procedures were: 30 mL pre-cultures were inoculated with *E. coli* BL21 (DE3) cells harboring pET28a(+) OLHR variants. These cultures were grown overnight in lysogeny broth (LB) medium, containing 30 µg/mL kanamycin. The pre-cultures were used to inoculate large cultures (1000 mL LB + 50 µg/mL kanamycin in 5 L shake flasks). Cells were grown at 37 °C, 180 rpm, until an OD_{600} between 0.6-0.8 was reached. Protein production was induced by the addition of 0.5 mM IPTG (final concentration) and the cells were left at 20 °C, 180 rpm, for overnight (18 hours). Cells were harvested by

centrifugation (11000 g at 4 °C for 10 min), washed with Tris-HCl buffer (50 mM, pH 7.5, 100 mM NaCl) and centrifuged again. The cell pellets were collected and stored at -80 °C for further use. In order to weigh the amount of the cells accurately, part of the cell pellets was lyophilsied overnight and solid powders were obtained. The amino acid sequence and DNA sequence of the enzyme is shown in Supplementary Figure 1.

Preparation of the 5,8-diol synthase from *Aspergillus nidulans* (*An*DS)

Plasmid construction

The recombinant pACYC-PelBSS-*An*DS, containing the signal sequence of *pelB* (PelBSS)-*An*DS gene fragment, was constructed by using the pET21a-AnDS, which had been constructed in an earlier study [3]. The gene encoding *An*DS was amplified by the polymerase chain reaction (PCR) using (pET-21a- *An*DS) as the template. Forward (5'-CCG GCG ATG GCC ATG GGT GAA GAC AAA GAA ACA AA-3') and reverse primers (5'-GGT GGT GGT GCT CGA GAA AAT CTT CCT TCA GTT GGG GCA-3') were synthesized by Cosmo Genetech (Korea). The amplified DNA fragment was purified using a PCR purification kit and was ligated into the Nco Ⅰ and Xho Ⅰ sites of the pET-26b vector with In-fusion HD Cloning kit (Takara). The resulting plasmid was used to construct pACYC-PelBSS-*An*DS. The gene encoding a PelB signal sequence, *An*DS, and His tag was amplified by the PCR using pET-26b-AnDS as the template. Forward (5'- AGG AGA TAT ACC ATG AAA TAC CTG CTG CCG ACC G -3') and reverse primers (5'- ATG CGG CCG CAA GCT TTA GTG GTG GTG GTG GTG G -3') were synthesized by Cosmo Genetech. The amplified DNA fragment was purified using a PCR purification kit and was ligated into the Nco Ⅰ and HindⅢ sites of the pACYCDuet-1 vector with In-fusion HD Cloning kit (Takara).

Preparation of the recombinant* E. coli *cells expressing the* An*DS

The *An*DS was expressed in *E. coli* BL21(DE3) by using the recombinant plasmids (i.e, pET21a-*An*DS [3] and pACYC-PelBSS-*An*DS). The recombinant *E. coli* cultures were grown overnight in terrific broth (TB) medium containing the appropriate antibiotics. The pre-cultures were used to inoculate large cultures (500 mL in 2 L shake flasks). The cells were grown at 37 °C, 180 rpm until an OD_{600} between 0.6-0.8 was reached. Protein production was induced by the addition of 0.1 mM IPTG and the cells were left at 16 °C, 150 rpm for overnight. The resulting cells were harvested by centrifugation and used as the biocatalysts.

Activity assay of the recombinant* E. coli *cells expressing the* An*DS

7 mg *E. coli* cells containing *An*DS (*An*DS @*E. coli*) and 3.4 mg of oleic acid were added into 980 µL of HEPS buffer pH 7.5 (50mM, with 10 % (v/v) DMSO). The resultant suspension was stirred at 40 °C for 1 to 2 h. To analyse the product, 3 mL of ethyl acetate (containing 5 g/L of palmitic acid as internal standard) was added to the above reaction suspension (3:1 volume ratio) and vigorously mixed. The organic phase was collected by centrifugation and was dried over $MgSO_4$. The obtained sample was analysed by gas chromatography/mass spectrophotometry (GC/MS) as previously reported[4, 5]. Notably, *E. coli* BL21(DE3) pACYC-PelBSS-*An*DS, expressing and sorting the *An*DS into the periplasm, displayed approximately 10-fold greater biotransformation rates and 2.4-fold higher final product (5,8-dihydroxy-9(Z)-octadecenoic acid) concentration in the reaction medium, as compared to the control strain *E. coli* BL21(DE3) pET21a-*An*DS, expressing the *An*DS in the cytoplasm (Supplementary Figure 25).

Preparation of *E. coli* BL21(DE3) pACYC-PelBSS-OhyA/pET28a-*Cv*FAP

The recombinant *E. coli* BL21(DE3) pACYC-PelBSS-OhyA/pET28a-*Cv*FAP co-expressing *Sm*OhyA and *Cv*FAP were grown overnight in TB medium, containing appropriate antibiotics. From these, 500 mL cultures (TB + appropriate antibiotics in 2 L shake flasks) were prepared (cell growth at 37 °C, 180 rpm, until an OD_{600} between 0.7-0.8 followed by induction by the addition of 0.5 mM IPTG). The cultures were incubated at 20 °C, 180 rpm, for another 20 h. Cells were harvested (centrifugation at 11000 g, 4 °C for 10 min) and resuspended directly into the Tris-HCl buffer (50 mM, pH 8, 100 mM NaCl) for biotransformation.

General procedures for cascade reactions

2.5-20 mg of lyophilised whole cells of oleate hydratase, and 2.0 mg of oleate acid were added into 980 µL of Tris-HCl buffer (100 mM, with 50 mM of NaCl). The resultant suspension was stirred at 30 °C for 11 hours. 20 µL of photodecarboxylase (from stock solution with a concentration of 102 µM) was added afterwards and the suspension was illuminated by blue LED and stirred for another 6 hours. The final reaction condition was: Reaction condition: [substrate]=7 mM, [lyophilised oleate hydratase whole cell]= 2.5-20 mg mL^{-1}, [*Cv*FAP]=2 µM, Tris-HCl buffer (pH 8.0, 100 mM, with 50 mM of NaCl), blue light (intensity=13.7 $mEL^{-1}s^{-1}$), total volume 1.0 mL. To analyse the product, 1.0 mL of ethyl acetate (containing 5 mM of 1-octanol) was added to the above reaction suspension (1:1 volume ratio) and vigorously mixed using a vortex mixer. The organic phase was collected by centrifuge and was dried over $MgSO_4$. The obtained sample was analysed by GC (Cp sil 5CB, column 50 m × 0.53 mm × 1.0 µm).

For the photoenzymatic diol synthesis-decarboxylation of oleic acid, 7 mg *E. coli* cells containing 5,8-diol synthase (*An*DS cells) and 7 mg of oleic acid were added into 980 µL of HEPES buffer pH 7.5 (50mM, with 10 % (v/v) DMSO). The resultant suspension was stirred at 40 °C for 2 h. Afterwards, 7 mg *E. coli* cells containing photodecarboxylase (*Cv*FAP cells) was added and the suspension was illuminated with blue LED light and stirred for another 7 h. The final reaction conditions were: [oleic acid] = 15 mM, [*An*DS cells] = 7 g L^{-1}, [*Cv*FAP cells] = 7 gL^{-1}, HEPES buffer pH 7.5 (50mM, with 10 % (v/v) DMSO), blue light (intensity=13.7 $mEL^{-1}s^{-1}$), total volume 1 mL. To analyse the product, 3 mL of ethyl acetate (containing 5 g/L of palmitic acid as internal standard) was added to the above reaction suspension (3:1 volume ratio) and vigorously mixed. The organic phase was collected by centrifugation and was dried over $MgSO_4$. The obtained sample was analysed by GC/MS as previously reported[4, 5].

For the photoenzymatic *Sm*OhyA-hydratation and *Cv*FAP-decarboxylation of oleic acid, recombinant *E. coli* BL 21 (DE3) pACYC-PelBSS-OhyA/pET28a-CvFAP was added into 50 mM Tris-HCl buffer (pH 6.5) containing 5 mM oleic acid. For the reaction, first the *Sm*OhyA-catalysed hydration reaction was performed for 0.125 h followed by *Cv*FAP-catalysed decarboxylation under illumination for another 1.625 h. The final reaction conditions were: Reaction condition: [oleic acid] = 5 mM, [*E. coli* co-expressing *Sm*OhyA and *Cv*FAP] = 7 gL^{-1}, Tris-HCl buffer pH 6.5 (50mM), illumination with blue light (λ = 450 nm; intensity = 13.7 $mEL^{-1}s^{-1}$.

Biphasic cascade reactions

In order to increase the solubility of oleic acid we used we used a so-called two liquid phase approach with triolein as organic phase serving both as substrate reservoir and product sink. 20 mM oleic acid was first dissolved in triolein. 500 µL of this stock solution was added to 480 µL of Tris-HCl buffer (pH 7.5, 100 mM, with 50 mM of NaCl) containing 10 mg of lyophilised whole cells of oleate hydratase. The reaction was continued for 24 hours at 30 °C. 20 µL of photodecarboxylase (from stock solution with a concentration of 102 µM) was added afterwards and the suspension was illuminated by blue LED and stirred for overnight (15 hours). The product was analysed via gas chromatography.

Trienzymatic cascade reactions transforming triglycerides into alcohols

We also envisioned a trienzymatic cascade by using lipase (from *Candida rugosa*) to hydrolyse triolein directly. To achieve this, 500 µL of triolein containing 0.5 mg of lipase and 480 µL of Tris-HCl buffer (pH 7.5, 100 mM, with 50 mM of NaCl) containing 10 mg of lyophilised oleate hydratase were mixed and stirred at 30 °C for 24 hours. Then the mixture was heated to 90 °C for 90 seconds. After cooling down to room temperature 20 µL of photodecarboxylase (from stock solution with a concentration of 102 µM) was added and the suspension was illuminated by blue LED and stirred for overnight (15 hours). The product was analysed via gas chromatography.

General procedure for the quantification of hydroxy fatty acids (2-11b):

In a 4 mL glass vial, 980 µL of Tris-HCl buffer (pH 8.0, 100 mM, with 50 mM of NaCl) containing 10 mM of substrate and 20 mg of lyophilised cells (overexpressing oleate hydratase) were mixed and stirred at 30 °C for 11 hours. At the end of the reactions, the mixture was extracted with deuterated chloroform ($CDCl_3$, 1 mL). The organic phase was dried over $MgSO_4$. The obtained sample was analysed by NMR. In order to determine the conversion of the reaction, ^1H NMR was used by using trimethoxybenzne (TMS) as an internal standard or integrating the characteristic peaks of the substrate (5.35 ppm from HC=CH) and (CH-OH) of the hydroxy fatty acids (intermediates).

General procedure for the quantification of fatty alcohols (2-11c):

In a 4 mL glass vial, 980 µL of Tris-HCl buffer (pH 8.0, 100 mM, with 50 mM of NaCl) containing 10 mM of substrate and 20 mg of lyophilised oleate hydratase cells were mixed and stirred at 30 °C for 11 hours. 20 µL of photodecarboxylase (from stock solution with a concentration of 102 µM) was added afterwards and the suspension was illuminated by blue LED and stirred for another 6 hours. At the end of the reactions, the mixture was extracted with deuterated chloroform ($CDCl_3$, 1 mL). The organic phase was dried over $MgSO_4$. The obtained sample was analysed by NMR. In order to determine the conversion of the product, ^1H NMR was used by using trimethoxybenzne (TMS) as an internal standard or integrating the characteristic peaks of the substrate (5.35 ppm for HC=CH) and (CH-OH) the fatty alcohols.

Preparative-scale synthesis starting from linoleic acid

In a 100 mL scale, 98 mL of Tris-HCl buffer (pH 8.0, 100 mM, with 50 mM of NaCl) containing 10 mM of substrate and 2 g of lyophilised oleate hydratase cells were mixed in a beaker and stirred at 30 °C for 48 hours. The beaker was sealed by using parafilm. 1 g of the lyophilized enzyme was added after 24 hours. 2 mL of photodecarboxylase (from stock solution with a concentration of 102 µM) was added afterwards and the suspension was illuminated by blue LED and stirred for 48 hours. 1 ml of the photodecarboxylase was added after 24 hours during the time course. The final reaction condition was: [linoleic acid] = 10 mM, [lyophilised oleate hydratase whole cells] = 20 mg mL^{-1}, [*Cv*FAP] = 2 µM, Tris-HCl buffer (pH 8.0, 100 mM, with 50 mM of NaCl), blue light (intensity=13.7 Me $L^{-1}s^{-1}$), total volume 100 mL.

At the end of the cascade reactions, the mixture was extracted with ethyl acetate (75 mL, 2×). The organic phase was combined. After the removal of ethyl acetate under reduced pressure, the product was purified via flash chromatography (liquid loading) on silica gel using heptane/ethyl acetate 40:1 as eluent for 15 min, followed by a programmed gradient for 10 min (ethyl acetate /heptane (2.5 to 80% ethyl acetate /heptane gradient). 82.5 mg (32.5% isolated yield) of the corresponding alcohol was obtained starting from linoleic acid.

Analysis using GC

The products starting from oleic acid and triolein were analysed and quantified by GC (model: SHIMADZU GC-2014) equipped with column Cpsil 5 CB: (50 m × 0.53 mm × 1.0 µm), FID, N2 as the carrier gas. Temperature program: 110 °C hold 3.4 min, 25 °C/min to 190 °C hold 2.1 min, 25 °C/min to 230 °C hold 4.1 min, 30 °C/min to 320 °C hold 3.0 min. Retention time (min) of the compounds: 1-octanol 3.22, heptadec-8-ene 8.74, 9-heptadecanol 10.61, oleic acid 12.51, 10-hydroxystearic acid 15.59.

Analysis using GS/MS

All products obtained from other unsaturated fatty acids (except oleic acid) in the reactions were analysed by GC/MS (model: SHIMADZU GC-2014) equipped with column Cpsil-5: (25 m × 0.25 mm × 0.4 µm).
In order to determine the structure of the fatty alcohols and hydroxyl fatty acids, silylation was performed. Typical procedures are: the 300 µL of the reaction mixture was extracted by using 300 µL of ethyl acetate. The organic phase was collected by centrifugation and was dried over $MgSO_4$ (2×). The organic phase was then evaporated by using N_2 flow, followed by the addition of 50 µL of *N*,*O*-bis(trimethylsilyl)trifluoroacetamide (BSTFA). After incubation at 100 °C for 60 min, the reaction mixture was cooled down and diluted with 1500 µL of ethyl acetate and analysed by GC/MS. In order to (semi)quantify the fatty alcohols, 9-heptadecanol were silylated in the same manner as described above and 1-octanol was used as internal standard. The peak area of the derivatised fatty alcohols were compared to that of silylated 9-heptadecanol from a calibration curve. The accuracy of this quantification method was validated by NMR quantification (with and without internal standard, Tables S2 and S3). It should be noted that, after the silylation process, eight additional peaks (5.980, 7.385, 8.391,

8.772, 10.16, 10. 220, 11.019, 12.34 min, respectively) appeared in GC chromatogram; they were observed very reproducibly. A careful investigation only using substrates confirmed that this phenomenon was due to artefacts from the trimethylsilyl derivatisation reactions. This has also been reported previously[7].

Concentrations of the *An*DS and *Cv*FAP-reaction substrates, intermediates, and products were measured as described previously[4, 5]. The reaction medium was mixed with a 3x volume of ethyl acetate containing palmitic acid as an internal standard. The organic phase was harvested after vigorous vortexing and was then subjected to derivatisation with N-methyl-N-(trimethylsilyl) trifluoroacetamide (TMS). The TMS-derivatives were analyzed by GC/MS (Agilent Technologies) equipped with a flame ionization detector and a split injection system (split ratio set at 1:10) and fitted with a nonpolar capillary column (30 m length, 0.25-µm thickness, HP-5MS, Agilent Technologies). Column temperature was increased from 90 to 255 °C at a rate of 5 °C/min, and then maintained at 255 °C. The injector and detector temperatures were 260 and 250 °C, respectively. Mass spectra were obtained by electron impact ionization at 70 eV. Scan spectra were obtained within the range of 100–600 m/z. Selected ion monitoring (SIM) was used for the detection and fragmentation analysis of the reaction products.

NMR measurements

The NMR analysis and determination of enantiomeric excess (e.e.) of fatty alcohols were performed on Agilent 400 MHz NMR spectrometer. In order to determine the e.e. the reactions were performed in 10 mL scale. At the end of the cascade reactions, dichloromethane (DCM, 5 mL, 3×) was added to extract the crude the products. The organic phase was combined and dried over $MgSO_4$ (2×). After the DCM was evaporated under reduced pressure, 1.5 mg of 4-(dimethylamino)pyridine (12.5 µmol), 15.5 mg of *N*,*N'*-dicyclohexylcarbodiimide (75 µmol), 14.6 mg of (*S*)-(+)-*O*-acetylmandelic acid (75 µmol) and 1 mL of anhydrous dichloromethane were added to the vial and magnetically stirred at room temperature for 3 hours. 20 µL of water was added and stirred for 30 min to quench the reaction[6]. The mixture was filtered and the filtrate was evaporated to dryness. The crude were dissolved in $CDCl_3$ for NMR analysis. The integration of the signal at 5.84-5.95 ppm was used to calculate the enantiomeric excess[7].

Quantification of the reagents was performed using ^{1}H NMR by integrating the characteristic peaks of the substrate (5.35 ppm from *H*C=C*H*) and the products (ca. 3.6 ppm, C*H*-OH for the hydroxy fatty acids and ca. 3.7 ppm, C*H*-OH for the final, decarboxylated products). The sample was prepared by directly extracting the reaction mixture using $CDCl_3$, dried over $MgSO_4$ and subjecting to ^{1}H NMR measurement.

Supplementary References

1. Zhang W, Ma M, Huijbers M, Filonenko GA, Pidko EA, van Schie M*, et al.* Hydrocarbon synthesis via photoenzymatic decarboxylation of carboxylic acids. *J. Am. Chem. Soc*. **141**, 3116–3120 (2019).

2. Huijbers M, Zhang W, Hollmann F. Light-driven enzymatic decarboxylation of fatty acids. *Angew. Chem. Int. Ed.* **57**, 13648-13651 (2018).

3. Seo M-J, Shin K-C, Jeong Y-J, Oh D-K. Production of 5,8-dihydroxy-9(Z)-octadecenoic acid from oleic acid by whole recombinant cells of *Aspergillus nidulans* expressing diol synthase. *Biotechnol. Lett.* **37**, 131-137 (2015).

4. Seo E-J, Kang CW, Woo J-M, Jang S, Yeon YJ, Jung GY*, et al.* Multi-level engineering of Baeyer-Villiger monooxygenase-based *Escherichia coli* biocatalysts for the production of C9 chemicals from oleic acid. *Metab. Eng.* **54,** 137-144 (2019).

5. Seo E-J, Yeon YJ, Seo J-H, Lee J-H, Boñgol JP, Oh Y*, et al.* Enzyme/whole-cell biotransformation of plant oils, yeast derived oils, and microalgae fatty acid methyl esters into n-nonanoic acid, 9-hydroxynonanoic acid, and 1,9-nonanedioic acid. *Bioresour. Technol.* **251**, 288-294 (2018).

6. Engleder M, Pavkov-Keller T, Emmerstorfer A, Hromic A, Schrempf S, Steinkellner G*, et al.* Structure-Based Mechanism of Oleate Hydratase from *Elizabethkingia meningoseptica*. *ChemBioChem* **16**, 1730-1734 (2015).

7. Engleder M, Strohmeier GA, Weber H, Steinkellner G, Leitner E, Müller M*, et al.* Evolving the Promiscuity of *Elizabethkingia meningoseptica* Oleate Hydratase for the Regio- and Stereoselective Hydration of Oleic Acid Derivatives. *Angew. Chem. Int. Ed.* **58**, 7480-7484 (2019).

8. Little JL Artifacts in trimethylsilyl derivatization reactions and ways to avoid them. *J. Chromatogr. A* **844**, 1-22 (1999).
